# Therapeutically actionable signaling node to rescue AURKA driven loss of primary cilia in *VHL*-deficient cells

**DOI:** 10.1038/s41598-021-89933-7

**Published:** 2021-05-17

**Authors:** Pratim Chowdhury, Dimuthu Perera, Reid T. Powell, Tia Talley, Durga Nand Tripathi, Yong Sung Park, Michael A. Mancini, Peter Davies, Clifford Stephan, Cristian Coarfa, Ruhee Dere

**Affiliations:** 1 Center for Precision Environmental Health, Baylor College of Medicine, One Baylor Plaza, BCM130, Houston, TX 77030 USA; 2 Department of Molecular and Cellular Biology, Baylor College of Medicine, Houston, TX USA; 3 Center for Translational Cancer Research, Institute of Biosciences and Technology, Texas A&M College of Medicine, Houston, TX USA

**Keywords:** Kidney diseases, Cell signalling, Phosphoinositol signalling

## Abstract

Loss of primary cilia in cells deficient for the tumor suppressor *von Hippel Lindau* (*VHL*) arise from elevated Aurora Kinase A (AURKA) levels. VHL in its role as an E3 ubiquitin ligase targets AURKA for degradation and in the absence of *VHL*, high levels of AURKA result in destabilization of the primary cilium. We identified NVP-BEZ235, a dual PI3K/AKT and mTOR inhibitor, in an image-based high throughput screen, as a small molecule that restored primary cilia in *VHL*-deficient cells. We identified the ability of AKT to modulate AURKA expression at the transcript and protein level. Independent modulation of AKT and mTOR signaling decreased AURKA expression in cells confirming AURKA as a new signaling node downstream of the PI3K cascade. Corroborating these data, a genetic knockdown of *AKT* in cells deficient for *VHL* rescued the ability of these cells to ciliate. Finally, inhibition of AKT/mTOR using NVP-BEZ235 was efficacious in reducing tumor burden in a 786-0 xenograft model of renal cell carcinoma. These data highlight a previously unappreciated signaling node downstream of the AKT/mTOR pathway via AURKA that can be targeted in *VHL*-null cells to restore ciliogenesis.

## Introduction

The *von Hippel Lindau* (*VHL*) tumor suppressor is causally linked to renal cell carcinoma (RCC) and VHL disease, characterized by the systemic development of benign and malignant lesions in multiple organs^1^. The molecular basis of VHL pathogenesis was directly related to its role as an E3 ubiquitin ligase that targets hypoxia inducible factor alpha (HIFα) for proteolytic degradation^2^. Oher non-HIF related functions of VHL have now been established, including its role in maintaining microtubule-based cytoskeletal elements such as the mitotic spindle^3,4^ and the primary cilium^5,6^.

The primary cilium is a microtubule-rich organelle that functions as a central integrator of extracellular signals that modulate a number of cellular processes^7,8^. In line with these functions, there are a diverse and complex network of inputs essential for formation, maintenance and functioning of this organelle^9^. Loss of *VHL* results in a corresponding loss of primary cilia categorizing VHL as a ciliopathy—a growing collection of diseases characterized by absent/aberrant primary cilia^10,11^. Studies in primary human RCC tumors confirmed the loss of primary cilia in tumor tissue compared to adjacent parenchyma^12^. Primary cilia are cell cycle dependent, formed in quiescence, and disassembled prior to cells entering mitosis. Recent studies establish the cilium as a checkpoint that prevented cells from re-entering the cell cycle^9^. Aurora Kinase A (AURKA) a well-established mitotic kinase is involved in cilia disassembly via its activation of HDAC6^13^. Other mitotic kinases such as Polo-like kinase 1 (Plk1)^14^ and NIMA related kinase 2 (Nek2)^15^ were also identified as negative regulators of ciliogenesis, thereby intricately linking the cell cycle to ciliogenesis.

Protein kinase B (PKB)/AKT activation occurs downstream of the phosphatidylinositol 3-kinase (PI3K) signaling pathway, which is a key regulator of survival during cellular stress and is often activated in many cancers^16^. The PI3K pathway is intricately entwined with the mammalian target of rapamycin (mTOR) signaling pathway which integrates multiple cellular inputs to regulate protein synthesis and growth^17–19^. Through its biological targets AKT regulates a wide-range of cellular processes including cell survival, proliferation and cell cycle, cytoskeletal organization and vesicular trafficking^19^ to name a few. Activation of the PI3K/AKT signaling pathway is common in human kidney cancers and in cysts arising from loss of *VHL*^20^. Conversely, inactivation of the negative regulator of this pathway—PTEN, is also reported in kidney cancer^21^. Activated AKT (phospho-AKT) localizes at the base of the primary cilium where it binds and phosphorylates a ciliary protein—inversin. Ciliary PI3K/AKT signaling was further regulated by the PI3K inhibitor inositol polyphosphate-5-phosphatase E (INPP5E), which also localized to the primary cilium^22^. Interestingly, INPP5E was phosphorylated by AURKA and conversely INPP5E also regulated AURKA activity in a complex relationship that linked AURKA to PI3K signaling.

Downstream of activated AKT, mTOR signaling is implicated in ciliogenesis. Previously, we reported that polycystin-1, a cilia-specific protein, regulated localization of tuberin (TSC2), upstream of mTORC1, to repress mTOR signaling^23^. Several upstream regulators of mTORC1 such as LKB1 and AMPK localize to the cilium. LKB1 activated AMPK signaling at the cilium in response to fluid flow resulting in the downregulation of the mTOR signaling cascade^24^. In addition, rapamycin, an inhibitor of mTOR signaling modulated cilia length and function^25^ further implicating a role for PI3K/AKT/mTOR signaling at the cilium.

Our previous work demonstrated that loss of cilia in *VHL*-deficient cells arises from elevated AURKA levels^26,27^. VHL in its role as an E3 ubiquitin ligase targeted AURKA for degradation^27^, and in *VHL*-null cells, elevated AURKA expression resulted in destabilization of the cilium. Recently, using an image-based high throughput assay we identified two potential small molecules that restored cilia formation in *VHL*-deficient cells^28^. Previously we reported bexarotene as a novel modulator of ciliogenesis via its ability to regulate AURKA^28^. In the present study we validate and confirm AKT as a bone fide modulator of primary cilia in cells. A dual PI3K/AKT and mTOR inhibitor, NVP-BEZ235, rescued cilia formation in *VHL*-null cells. We found that AKT regulated AURKA expression at the mRNA transcript and protein level independent of VHL-status. A strategy of genetically knocking down AKT in the setting of *VHL*-deficiency successfully increased the percentage of ciliated cells. Inhibition of AURKA and the PI3K/mTOR pathway using alisertib and NVP-BEZ235, respectively reduced tumor burden in a 786-0 xenograft model of RCC. These data highlight the inherent complexity of VHL pathogenesis and present a strategy wherein co-targeting PI3K/AKT, mTOR and AURKA could have potential value in a clinical setting of RCC.

## Results

### Identification of NVP-BEZ235 as a positive regulator of primary cilia in *VHL*-deficient cells

**Figure 1:**
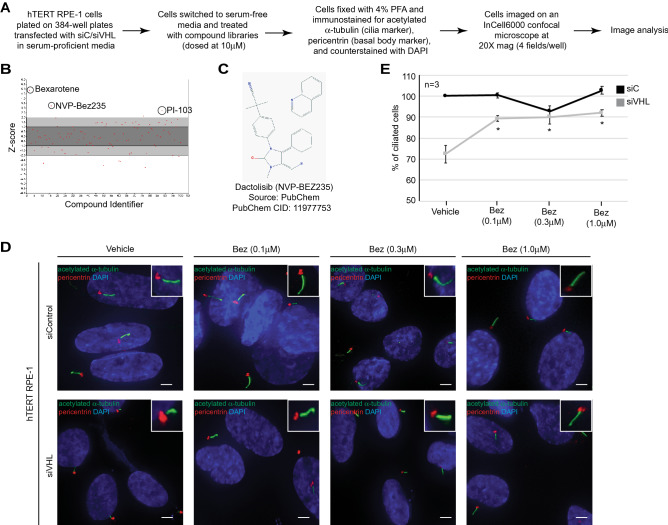
NVP-BEZ235 regulates ciliogenesis in *VHL*-deficient cells. (**A**) Workflow used for the image-based screening assay. (**B**) Graphical representation showing the Z-score of compounds used in the screen. The compounds annotated with names were identified as positive hits. (**C**) Chemical structure of NVP-BEZ235. (**D**) Representative images (60X magnification) of hTERT RPE-1 cells transfected with siC (scrambled control) or siVHL showing acetylated α-tubulin (cilia marker, green) and pericentrin (basal body marker, red). Nuclei are counterstained with DAPI in blue. Insets show a magnified image of a cilium for each of the treatment conditions. Scale bar, 10 μM. (**E**) Graphical representation of the percentage of ciliated cells treated with Vehicle or NVP-BEZ235 (Bez) at the indicated doses in *VHL*-proficient (siC, black line) and *VHL*-deficient (gray line) cells. *Statistical significance (Students t-test) compared to vehicle (DMSO) treated control (p < 0.01).

We recently developed a high throughput screening (HTS) assay to identify small molecules capable of restoring primary cilia in *VHL*-deficient cells^28^. This assay was established with a dual goal of identifying novel therapeutic targets and signaling pathways that directly modulate ciliogenesis in the setting of VHL disease and renal cell carcinoma (RCC). Our previously described image-based primary screen^28^ yielded a robust Z′ value of 0.62 and is summarized in the schematic shown in Fig. 1A. Positive hits were identified by computing Z-scores, and compounds 2 standard deviations (95% confidence interval) above the population mean were selected as positive ‘hits’. However, given that we identified two compounds that were 3 standard deviations (99% confidence interval) above the population mean (Fig. 1B), we prioritized these compounds for further validation in secondary assays. We identified bexarotene (described earlier)^28^ and NVP-BEZ235 as positive regulators of primary cilia in *VHL*-deficient hTERT RPE-1 cells (Fig. 1B). NVP-BEZ235 (dactolisib) is an imidazoquinoline derivative (Fig. 1C), which acts as a dual ATP-competitive phosphatidylinositol 3 kinase (PI3K) and mammalian target of rapamycin (mTOR) inhibitor^29^.

Given that NVP-BEZ235 yielded a mean Z-score of 3.2 (Fig. 1B), we moved NVP-BEZ235 into secondary validation dose response studies to confirm its ability to modulate cilia in the setting of *VHL*-deficiency. These assays were performed using freshly procured compound from an alternate source compared to that used for the primary screen. Consistent with our observations from the primary screen, treatment of *VHL*-deficient cells with three low-doses of NVP-BEZ235 (0.1 μM, 0.3 μM, and 1.0 μM) rescued the ability of these cells to ciliate (Fig. 1D). The percentage of ciliated cells was quantitated using a custom MATLAB analysis algorithm to identify and segment cilia and their adjoining basal bodies. Although the primary screen described previously^28^ applied a Pipeline Pilot derived customized image analysis algorithm, restrictions associated with the proprietary license, forced us to ultimately generate a more broadly applicable analysis algorithm. Briefly, the cilia and basal bodies were defined and segmented using maximum pixel intensity and size thresholds. Cilia were further delineated by setting the maximum allowable distance between the basal body and cilium to 12 pixels. A distance greater than 12 pixels resulted in a cilium that was excluded from further analysis, similar to the presence of multiple basal bodies in close (12 pixel) vicinity (usually associated with overlapping cilia) that were also excluded from further analysis. The length of the cilium was measured as half of the length of the perimeter drawn along the entire cilium. A ‘thinning operation’ was further applied to the cilium which thins the target object, in this case the cilium, to its bare skeleton. Data obtained from these analyses revealed that NVP-BEZ235 rescued the inability of *VHL*-deficient cells to ciliate, showing a 27% increase in ciliated cells compared to vehicle control (Fig. 1E). The ability of all three NVP-BEZ235 doses (0.1 μM, 0.3 μM, and 1.0 μM) to rescue ciliation in *VHL*-deficient cells was comparable (Fig. 1D,E). These data established NVP-BEZ235 as a *bone fide* positive regulator of the primary cilium in *VHL*-deficient cells.

### NVP-BEZ235 modulates AURKA expression to rescue ciliogenesis in the setting of *VHL* loss

**Figure 2:**
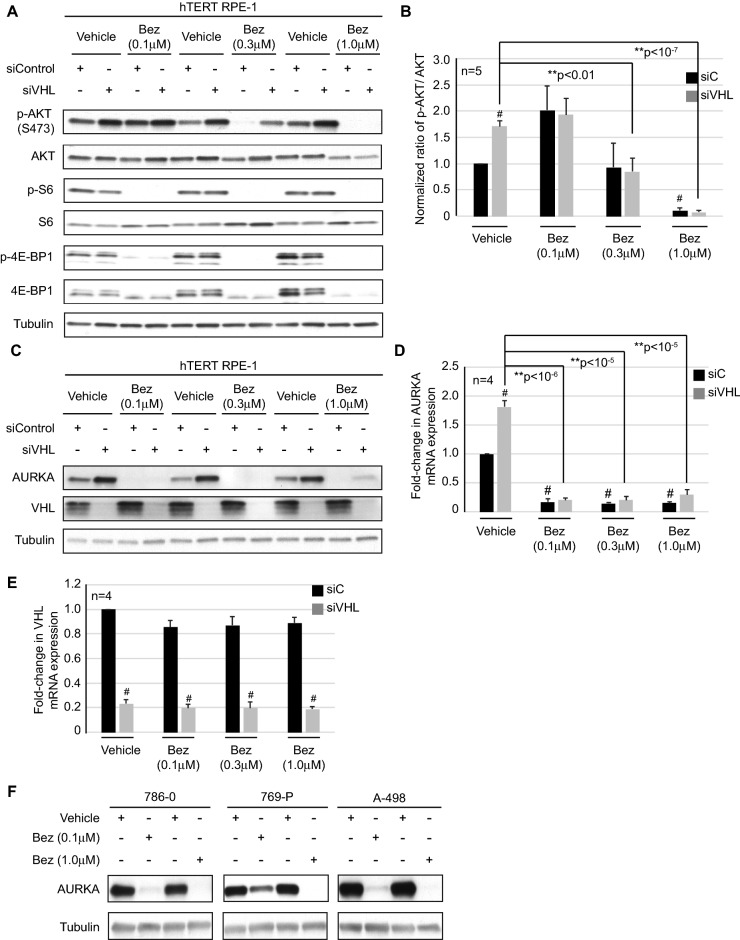
NVP-BEZ235 modulates AURKA expression in cells deficient for *VHL*. (**A**, **C**) *VHL*-proficient and *VHL*-deficient cells were treated with Vehicle or NVP-BEZ235 (Bez) as indicated and blotted for the antibodies shown. (**B**) Densitometric analysis graphed to show the ratio of phospho-AKT to total AKT. Black bars—siC (scrambled control) and gray bars—siVHL. ^#^Statistical significance (Students t-test) compared to vehicle (DMSO) treated control (siC) (p < 0.001), **statistical significance (Students t-test) compared to vehicle treated siVHL (p-values as indicated). (**D**) Graphical representation of densitometric data showing fold-change in AURKA mRNA expression. Black bars—siC and gray bars—siVHL. ^#^Statistical significance (Students t-test) compared to vehicle (DMSO) treated control (siC) (p < 0.001), **statistical significance (Students t-test) compared to vehicle treated siVHL (p-values as indicated). (**E**) Graphical representation of densitometric data showing fold-change in VHL mRNA expression. Black bars—siC and gray bars—siVHL. ^#^Statistical significance (Students t-test) compared to siC for each of the treatment conditions (p < 0.05). (**F**) Three *VHL*-null RCC cells lines (786-0, 769-P, A-498) treated with Vehicle and NVP-BEZ235 (Bez) at the indicated doses and blotted as shown.

Given that NVP-BEZ235 is a dual ATP-competitive phosphatidylinositol 3 kinase (PI3K)/protein kinase B (PKB/AKT) and mammalian target of rapamycin (mTOR) inhibitor^29^, we evaluated phosphorylation and activation of AKT and mTOR targets—S6 and 4E-BP1 as surrogates for treatment efficacy. Although, phospho-AKT (S473) levels decreased significantly in *VHL*-proficient and deficient cells treated with NVP-BEZ235 at the 0.3 μM and 1.0 μM dose (Fig. 2A and quantified in Fig. 2B), mTOR targets, phospho-S6 and phospho-4E-BP1, showed a significant decrease across all treatment doses (Fig. 2A), indicating the efficacy of NVP-BEZ235 in inhibiting PI3K/mTOR signaling in our study.

We recently established a direct linkage between loss of *VHL* and elevated AURKA expression, arising from the inability of the VHL E3 ligase to ubiquitinate and degrade AURKA^27^. Given that loss of primary cilia in *VHL*-null cells was causally linked to high AURKA expression^26,27^, we assessed levels of AURKA in *VHL*-deficient cells where primary cilia were rescued by treatment with NVP-BEZ235 (Fig. 1D,E). We observed a consistent and dramatic reduction in AURKA protein expression following treatment with NVP-BEZ235 at all doses (0.1 μM, 0.3 μM, and 1.0 μM), in both the scrambled control (siC) and siVHL transfected cells, compared to vehicle controls (Fig. 2C). Importantly, we found an analogous tenfold decrease in AURKA mRNA transcript levels in cells treated with NVP-BEZ235 at these doses (Fig. 2D). VHL transcript (Fig. 2E) and protein levels (Fig. 2C) were evaluated to confirm that changes in AURKA expression did not arise from increased VHL, which remained consistently low (siVHL) in both vehicle and treatment groups. Next, a panel of *VHL*-null renal cell carcinoma (RCC) cell lines with elevated AURKA expression^26,27^ were used to determine if NVP-BEZ235 treatment decreased AURKA expression. The *VHL*-null RCC cell lines (786-0, 769-P, A-498) showed a significant decrease in AURKA protein expression following treatment with NVP-BEZ235 (0.1 μM and 1.0 μM) (Fig. 2F) validating the data generated using an acute knockdown (siRNA) of *VHL*.

Next, we assessed if AURKA expression was transcriptionally modulated downstream of AKT by GSK3β. Phosphorylation and inactivation of GSK3β occurs downstream of AKT and AURKA (both kinases are able to phosphorylate GSK3β at S9 to inhibit its activity, in addition to other kinases) and given that GSK3β inactivation could further modulate AURKA mRNA transcript levels in *VHL*-deficient cells via β-catenin^26^, we assessed phospho-GSK3β (S9) levels. Treatment with NVP-BEZ235 increased phosphorylation of GSK3β (S9) in *VHL*-deficient cells (Supplemental Fig. S1A,B) and *VHL*-null RCC cell lines (Supplemental Fig. S1C,D) suggesting that NVP-BEZ235 failed to inhibit AURKA expression by reliving the inhibition on GSK3β. In summary, we show that treatment of *VHL*-deficient cells with NVP-BEZ235 results in decreased AURKA expression which likely correlates directly to the rescue of primary cilia in these cells.

### AKT and mTOR signaling modulates AURKA expression

**Figure 3:**
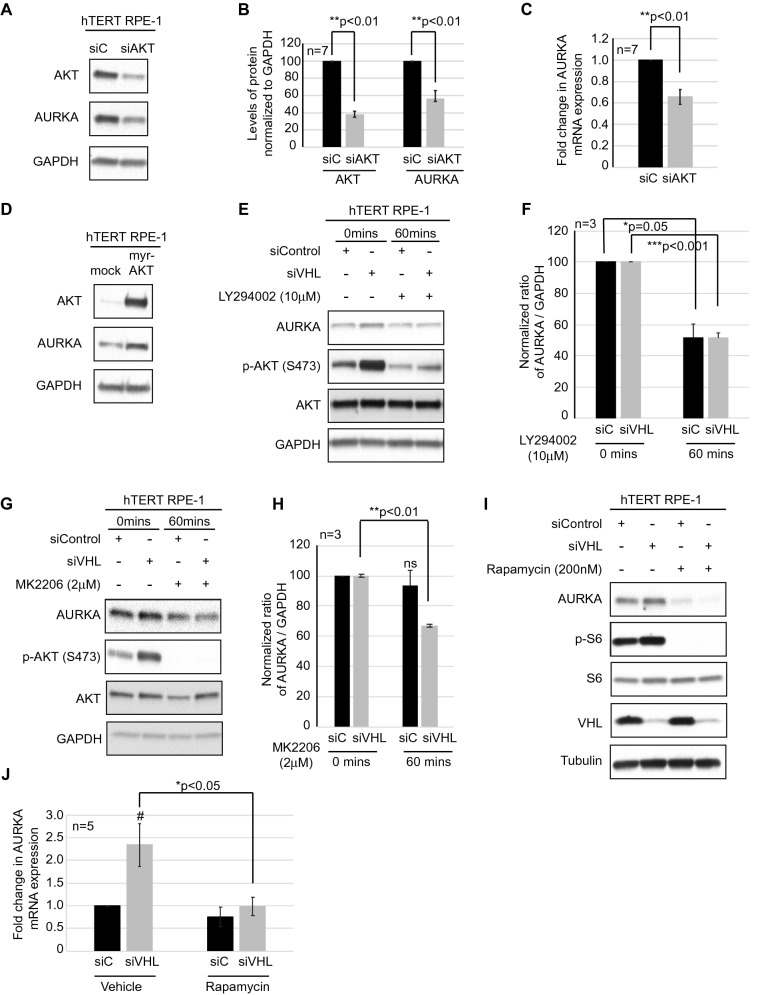
AKT and mTOR modulate AURKA. (**A**) hTERT RPE-1 cells proficient or deficient for *AKT* were blotted for the antibodies shown. (**B**) Graphical representation of densitometric data shows levels of AKT and AURKA normalized to 100. Black bars—siC (scrambled controls), gray bars—siAKT. (**C**) Graphical representation of AURKA mRNA transcript levels in hTERT RPE-1 cells transfected with siC (black bars) and siAKT (gray bars). **Statistical significance (Students t-test) with indicated p-values. (**D**) hTERT RPE-1 cells transfected with myristoylated (myr) AKT and blotted as indicated. (**E**, **G**) Whole cell extracts from hTERT RPE-1 cells proficient or deficient for *VHL* treated with LY294002 (10 μM) (**E**) or MK2206 (2 μM) (**G**) and blotted for the antibodies shown. (**F**, **H**) Densitometric values from immunoblots were graphed to show the normalized levels of AURKA protein levels in cells treated with LY294002 (**F**) or MK2206 (**H**). **^,^***Statistical significance (Students t-test) with p-values as indicated. (**I**) Whole cell extracts from hTERT RPE-1 cells proficient or deficient for *VHL* treated with rapamycin (200 nM) as indicated were blotted for the antibodies shown. (**J**) Graphical representation of AURKA mRNA transcript levels in hTERT RPE-1 cells transfected with siC (black bars) and siVHL (gray bars) and treated with rapamycin. ^#^Statistical significance (Students t-test) compared to siC (p < 0.05), *statistical significance (Students t-test) with indicated p-value.

To evaluate the contribution of PI3K signaling in modulating AURKA and to rule out any off-targets contributions of NVP-BEZ-235, AURKA expression was assessed in cells wherein we modulated AKT expression and activity. First, AKT was knocked down in hTERT RPE-1 cells using an siRNA strategy, which resulted in decreased AURKA expression both at the protein (Fig. 3A and quantified in Fig. 3B) and mRNA level (Fig. 3C). Overexpression of a constitutively active AKT construct (myristoylated (myr) AKT) in hTERT RPE-1 cells resulted in increased AURKA expression (Fig. 3D), further validating a role for AKT in modulating AURKA expression. Next, to determine if elevated AURKA expression could be rescued in the setting of *VHL*-deficiency we used two pharmacologic inhibitors of PI3K/AKT in *VHL*-proficient and deficient cells; LY294002 (PI3K inhibitor) and MK2206 (AKT inhibitor). Given that AKT signaling is rapidly (within minutes) modulated by these inhibitors and is not sustained over the 48 h time course required to ciliate cells, we chose to exclusively assess the ability of AKT to modulate AURKA expression within 60 min of treatment with these pharmacologic compounds without assessing ciliation in these cells. We observed a significant decrease in AURKA protein levels with LY294002 and MK2206 showing a 50% and 30% decrease in AURKA, respectively (Fig. 3E,G, quantified in Fig. 3F,H), although we failed to observe a corresponding decrease in AURKA mRNA transcripts 60 min post treatment (Supplemental Fig. S2A,B). Phospho-AKT (S473) levels, reduced dramatically and served to validate treatment efficacy. Next, to determine if modulation of mTOR activity, independent of PI3K signaling, also contributed to regulation of AURKA expression, we treated hTERT RPE-1 cells with rapamycin (mTOR inhibitor). Treatment of *VHL*-proficient and deficient cells with rapamycin significantly reduced AURKA expression at the protein (Fig. 3I), and mRNA levels (Fig. 3J). These data predict that independent modulation of either AKT or mTOR signaling can regulate AURKA highlighting an unexplored pathway downstream of PI3K dysregulation in RCC.

### Loss of *AKT* in *VHL*-deficient cells restores the ability of cells to ciliate

**Figure 4:**
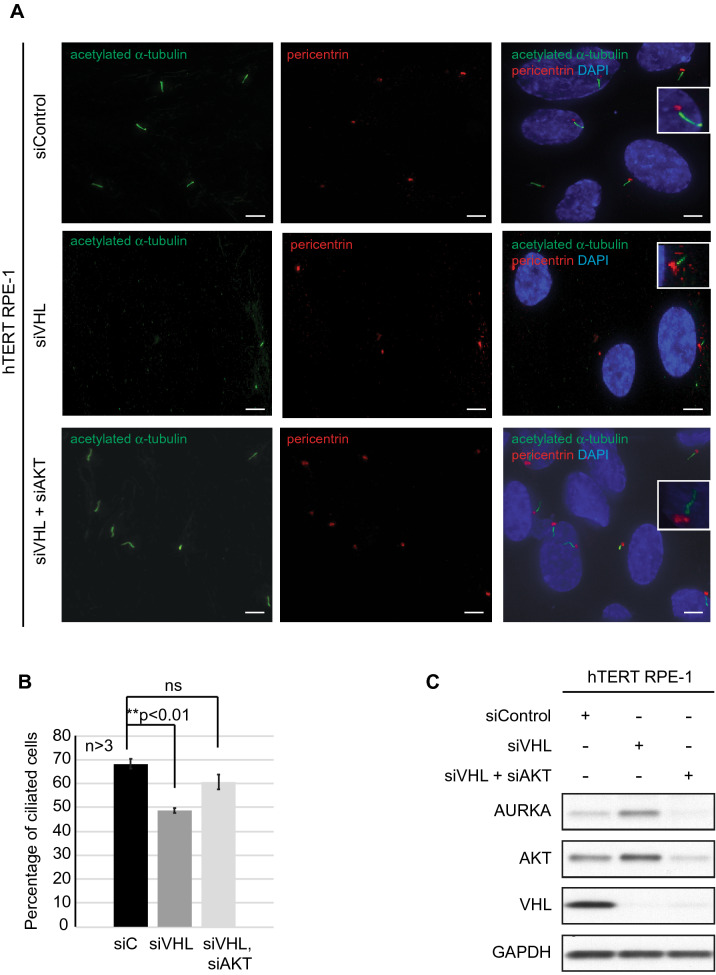
Co-deletion of *AKT* and *VHL* rescues ciliogenesis. (**A**) Representative images of hTERT RPE-1 cells (60X magnification) transfected with siC (scrambled control), siVHL or co-transfected with siVHL and siAKT showing acetylated α-tubulin (cilia marker, green) and pericentrin (basal body marker, red). Nuclei are counterstained with DAPI in blue. Insets show a magnified image of a cilium for each of the treatment conditions. Scale bar, 10 μM. (**B**) Graphical representation of the percentage of ciliated cells for each of the treatments as indicated in *VHL*-proficient (siC, black line) and *VHL*-deficient (gray line) cells. **Statistical significance (Students t-test) compared to vehicle (DMSO) treated controls with indicated p-values. (**C**) Whole cell extracts from hTERT RPE-1 cells transfected with siC, siVHL or co-transfected with siVHL and siAKT, immunoblotted using antibodies shown.

Given that PI3K signaling modulated AURKA expression, we assessed the ability of AKT to modulate AURKA activity. Primary cilia were evaluated as direct surrogates for AURKA’s biological activity (decreased AURKA activity correlates to increased ciliation) in hTERT RPE-1 cells by simultaneously knocking down both *AKT* and *VHL* (siVHL, siAKT). Consistent with a role for AKT in modulating AURKA activity, knocking down AKT restored the ability of *VHL*-null cells to ciliate (Fig. 4A) confirming a role for AKT signaling in regulating AURKA activity. We observed a 22% increase in ciliation in hTERT RPE-1 cells with a dual knockdown of *VHL* and *AKT* (siVHL, siAKT) compared to cells deficient for *VHL* alone (siVHL) (Fig. 4A,B). This restoration in the number of ciliated cells was comparable to levels that were observed in scrambled (siC) control cells (Fig. 4A,B). Next, we assessed AURKA levels in these cells to evaluate if loss of *AKT* in *VHL*-null cells rescued ciliogenesis by modulating AURKA expression. Corroborating our data in cells treated with NVP-BEZ235, we observed decreased AURKA expression in cells with a dual knockdown of *VHL* and *AKT* compared to cells with a monogenic loss of *VHL* (Fig. 4C). Together, these data suggest that AKT modulates AURKA expression and activity.

### Combined inhibition of AKT/mTOR and AURKA reduces tumor burden in a mouse xenograft model of RCC

**Figure 5:**
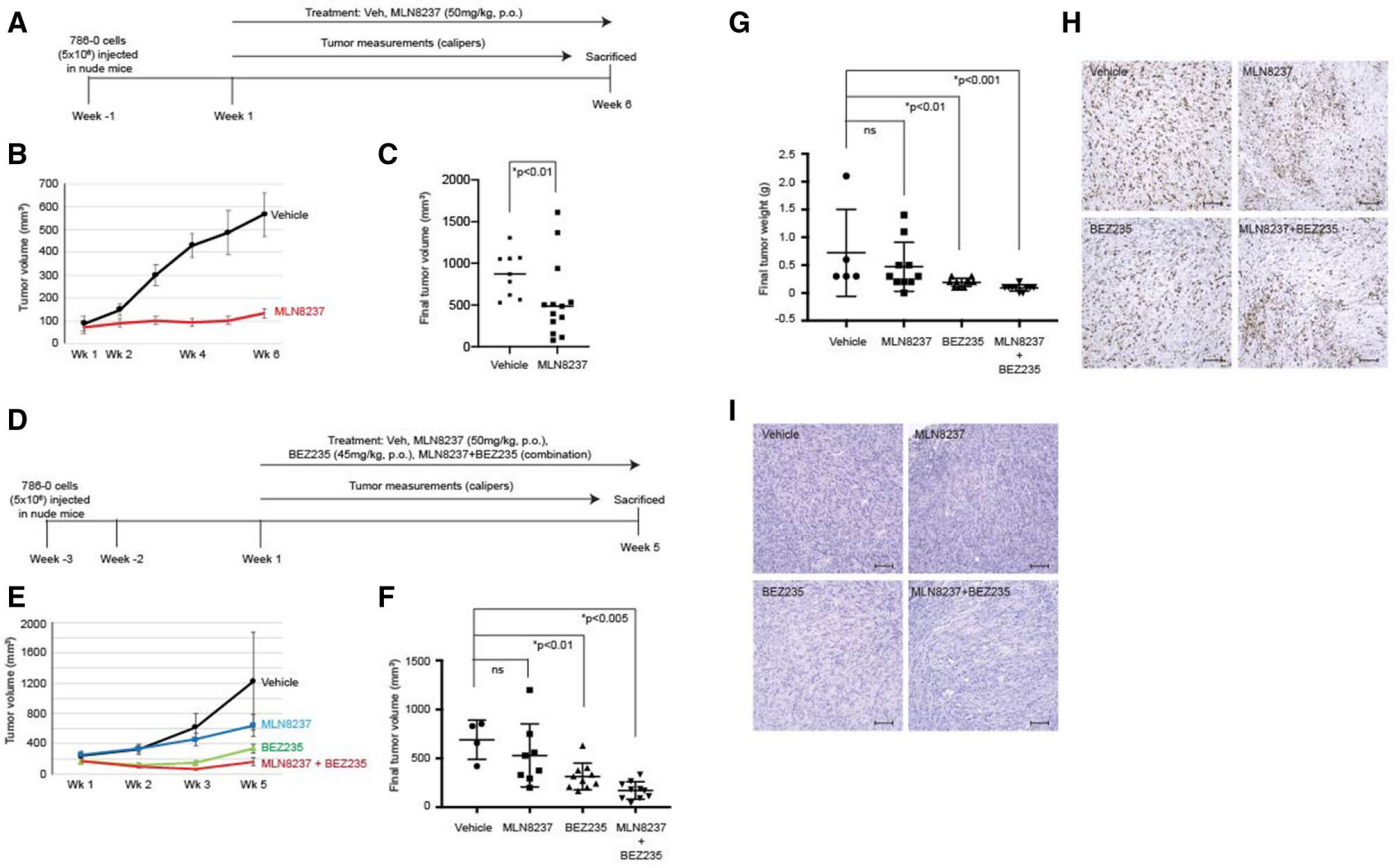
Combined inhibition of AKT/mTOR and AURKA signaling reduces tumor burden in a mouse xenograft model of RCC. (**A**) Schematic showing study design using a 786-0 mouse xenograft model treated with vehicle, and MLN8237. (**B**) Line chart plotted to show tumor volume (Week 1–Week 6). Vehicle (black circles), MLN8237 (red squares). (**C**) Scatter plot depicting the final tumor volume (mm^3^) from mice treated as indicated. *Statistical significance (Kolmogorov–Smirnov test) with p-value as indicated. (**D**) Schematic showing study design using a 786-0 mouse xenograft model treated with Vehicle, MLN8237, NVP-BEZ235 or a combination of MLN8237 and NVP-BEZ235. (**E**) Line chart plotted to show tumor volume (Week 1–Week 5, Week 4 the tumors were not measured and therefore not plotted). Vehicle (black circles), MLN8237 (blue squares), NVP-BEZ235 (BEZ235) (green triangle) and a combination of MLN8237 and NVP-BEZ235 (red line). (**F**, **G**) Scatter plot depicting the final tumor volume (mm^3^) (**F**) and final tumor weight (g) (**G**) from mice treated as indicated. *Statistical significance (Kolmogorov–Smirnov test) with p-values as indicated. (**H**, **I**) Representative images showing staining from tumor tissue for each of the treatments using Ki67 (**H**) and H&E (**I**) imaged at ×10 magnification. Scale bar, 500 μM.

786-0 *VHL*-null RCC cells are a great model for monitoring tumor growth in vivo, however one of the major limitations of this system is the inability of these cells to ciliate, even on re-expression of VHL as we have previously reported^28^. Moreover, monogenic *VHL* mouse models fail to exhibit a renal phenotype and co-deletion of multiple other tumor suppressors on chromosome 3p are known to be required to elicit a phenotype. Nonetheless, given that treatment of RCC cell lines, with elevated AURKA expression, using NVP-BEZ235 showed decreased AURKA expression (Fig. 2F), we wanted to correlate this decrease in AURKA expression to reduced tumor burden in an in vivo model of RCC. First, in a two-arm study using *VHL*-null RCC 786-0 cells we established xenografts in athymic nude mice (*Foxn1*^*nu*^). Mice were randomly divided into two arms and as shown in the schematic in Fig. 5A, a week following sub-cutaneous injection of the 786-0 cells the mice were treated with vehicle (Arm 1, n = 9) or MLN8237 (Arm 2, n = 13, 50 mg/kg, p.o.). Tumors were measured weekly for 6 weeks, where animals in the vehicle group showed a steady increase in tumor volume (Fig. 5B,C). Mice treated with MLN8237 did not develop fulminant tumors, with tumor volumes significantly reduced compared to control vehicles (Fig. 5B,C). Next, since our data implicated a role for AKT/mTOR signaling in modulating AURKA we designed a four-arm study using vehicle, MLN8237 (AURKA inhibitor, alisertib), NVP-BEZ235 (AKT/mTOR signaling inhibitor), and a combination of MLN8237 and NVP-BEZ235. Xenografts were established using 786-0 cells and mice randomly divided into four arms. In contrast to our data above treating mice with MLN8237, wherein we treated mice a week after sub-cutaneous injection of the cells we chose to treat mice with the pharmacologic agents when the tumors were palpable and averaged 200 mm^3^. As shown in the schematic in Fig. 5D, 4 weeks after sub-cutaneous injection of 786-0 cells into the flanks of the athymic mice, and when tumors averaged ~ 200 mm^3^, the animals were treated with vehicle (Arm 1, n = 5), MLN8237 (Arm 2, n = 10, 50 mg/kg, p.o), NVP-BEZ235 (Arm 3, n = 10, 45 mg/kg, p.o.), MLN8237 + NVP-BEZ235 (Arm 4, n = 10). Animals in the vehicle group showed a steady increase in tumor volume over the 5-week treatment period (Fig. 5E,F). Mice treated with MLN8237 as a single agent did not exhibit a significant decrease in tumor burden, although NVP-BEZ235 as a single agent was successfully able to decrease tumor burden (Fig. 5E–G). Importantly, a combination of MLN8237 and NVP-BEZ235 also significantly decreased tumor incidence, tumor volume and final tumor weight in the xenograft model of 786-0 (Fig. 5E–G). 80% of the mice treated with the combination (MLN8237 and NVP-BEZ235) showed a significant decrease in the size of the nodules/lesions to < 200 mm^3^ compared to the single agent treatments with NVP-BEZ235 and MLN8237 wherein only 20% and 10% of the mice, respectively, showed a reduction in size of lesions to < 200 mm^3^ (Fig. 5F,G). Although the tumor incidence rates and tumor volumes vastly differed between treatment groups, most importantly once the tumors did present the rate of proliferation, assessed using Ki67, was identical across all treatment groups (Fig. 5H). Not surprisingly, immunohistochemical analysis of tumor tissue using H&E from the treatment groups did not exhibit any differences compared to vehicle controls (Fig. 5I). These data suggest that co-inhibition of AURKA and AKT/mTOR signaling is successful in reducing tumor burden, providing proof-of-concept for future studies exploring efficacious compounds that target these pathways.

## Discussion

Loss of the tumor suppressor *VHL* is causally linked to renal cell carcinoma (RCC), and we and others have demonstrated the loss of primary cilia in *VHL*-deficient cells^26,27^. Recently, we identified two small molecules, in an image-based screening assay, that restored the ability of *VHL*-deficient cells to ciliate. In this study we confirmed and validated, PI3K signaling as a bone fide modulator of ciliogenesis^28^. Inhibition or loss of *AKT* restored the ability of *VHL*-null cells to ciliate, arising in part due to the downregulation of AURKA in these cells. AURKA in a non-mitotic role was found to activate HDAC6 and result in destabilization of the primary cilium^13^. Treatment with an AKT/mTOR inhibitor, NVP-BEZ235 (dactolisib), lead to a dramatic reduction in AURKA transcript and protein expression. Importantly, genetic and pharmacologic manipulations of AKT regulated AURKA expression. These data implicate a role for PI3K signaling in modulating ciliogenesis and AURKA activity at the cilium. Finally, a combination of an AURKA inhibitor, MLN8237 (alisertib) with NVP-BEZ235 in a 786-0 *VHL*-null mouse xenograft model of RCC dramatically reduced tumor burden, highlighting the potential for co-inhibition of AKT, mTOR and AURKA as a clinical intervention strategy.

The importance of PI3K/AKT and mTOR signaling at the primary cilium is only just beginning to emerge. Recently, AKT was found at the base of cilia in neural cells and pre-adipocytes^30,31^ with negative regulators of the PI3K/AKT signaling pathway, such as INPP5E also localizing at the cilium^32^. Cilia-specific INPP5E regulated a cilia-compartmentalized PI3K signaling axis, wherein loss of INPP5E and the resulting hyperactivation of AKT resulted in loss of primary cilia^32^. A combined deletion of *VHL* and *PTEN* also resulted in hyperactivation of the PI3K/AKT pathway and loss of primary cilia^20^. This loss of primary cilia associated with hyperactivation of AKT was linked to phosphorylation and inactivation of GSK3β, arising from increased AKT activity^20^. Our data support these observations and provide an alternate rationale of elevated AURKA activity downstream of AKT that prevents cilia formation. Previous studies also implicated GSK3β in maintaining the primary cilium^33^ with inactivation of GSK3β linked to loss of the cilium^20^. Recently, we showed that elevated AURKA expression in *VHL*-null cells arose from an inability of cells to degrade AURKA in the absence of VHL’s E3 ubiquitin ligase activity^27^ and was in part a direct consequence of increased β-catenin driven transcription of AURKA^26^. Increased AURKA expression in turn contributed to the phosphorylation and inactivation of GSK3 β (S9) in *VHL*-null cells^26^. Although, NVP-BEZ235 successfully inhibited both AKT and mTOR signaling and decreased AURKA, it failed to activate GSK3β in our study. Elevated levels of the inactivating phosphorylation on GSK3β (S9) observed in cells treated with NVP-BEZ235 could in part be attributed to upregulation of other kinases that also phosphorylate this residue or from off-target effects of NVP-BEZ235.

Importantly, there was a dramatic decrease in AURKA transcript and protein levels at the lowest dose of NVP-BEZ235, wherein we failed to observe AKT inhibition. This apparent discrepancy could be explained by decreased mTOR signaling which our data showed was independently able to modulate AURKA expression. Although AURKA’s role in regulating mTOR signaling via activation of AKT is well appreciated^34,35^, the mechanism of how mTOR signaling in turn could be modulating AURKA expression is as yet unclear. Recently, PI3K signaling was shown to regulate the abundance of its upstream activator AURKA through MYC-mediated transcription of AURKA^36^. Genetic alterations harboring MYC are common in RCC with focal amplifications of MYC-harboring regions validated in ccRCC^37,38^, although the role of MYC in RCC pathogenesis itself is complex with HIF2α and HIF1α playing opposing roles in modulating MYC transcriptional activity^39,40^. Interestingly, mTOR was found to dictate nucleocytoplasmic shuttling of GSK3β (independent of S9 phosphorylation) with inhibition of mTOR resulting in nuclear localization of GSK3β where it inhibited MYC^41^. Thus, it is likely that the decrease in AURKA observed in cells treated with NVP-BEZ235 could result from decreased MYC activation and subsequent reduced transcription of AURKA in the setting of mTOR inhibition.

A survey of more than 400 RCC tumors revealed significant and recurrent mutations in the PI3K/AKT pathway^37^ which promote both angiogenesis and proliferation. Given that PI3K/AKT/mTOR signaling was activated in RCC patients, rapalogues were FDA approved for use in the clinic, however, these agents showed tumor regression in a minority of patients with the development of resistance being a major limitation of therapy. This was in part attributed to the feedback activation of PI3K/AKT signaling^42,43^, although more recent work suggested that PI3K or AKT activation via feedback activation may be uncommon in RCC and may not be a major contributor to resistance^44^. Nonetheless, AKT and mTOR signaling is activated in RCC and has been targeted with rapalogues. However, elevated AURKA expression was found to limit the efficacy of PI3K/AKT targeted therapy and confer drug resistance in a number of cell lines^36^. Mapping of kinome dynamics revealed that incomplete inhibition of AURKA was a common source of therapy failure resulting from activation of AKT and residual mTOR activation^36^. Our data highlight the complex interplay between these pathways and provides a rationale for targeting both these signaling nodes which could be beneficial and perhaps even necessary in the setting of *VHL* loss.

NVP-BEZ235, an orally available PI3K, mTORC1 and mTORC2 inhibitor, was primarily used as a tool compound in our studies to identify the underlying pathways that regulated cilia formation in *VHL*-deficient cells. The reasoning for using NVP-BEZ235 as a tool compound as opposed to pursuing it as a lead compound, stemmed from early termination of clinical trials in RCC due to poor tolerability and modest clinical activity^45^ despite the promising preclinical studies in breast^46^, colon^47^, liver^48^ and other cancer models using this compound. Nonetheless, the strong rationale of inhibiting both PI3K and mTOR signaling has led to the continued search for alternate compounds that can inhibit AKT and mTOR signaling (ideally inhibiting both mTORC1 and mTORC2 complexes). Recently, GNE-477 another small molecule was found to have efficacy in inhibiting RCC growth and proliferation in vitro and in preclinical xenograft models^49^. We found that AURKA inhibition as a single agent in a mouse xenograft model of RCC had a modest effect on reducing tumor burden when tumors were already palpable, but was competent in preventing tumors from taking when treated at an earlier time point. These data suggest that the timing of AURKA inhibition should be a critical factor evaluated in future studies and perhaps exclusively targeting AURKA might be insufficient at reducing tumor growth, further emphasizing the importance of targeting multiple synergistic pathways in any given tumor. The applicability of single agents however would not be realistic or feasible for multiple reasons including development of resistance and the toxicity associated with these inhibitors, however our data provide proof-of-concept that targeting multiple pathways, in this case AURKA and PI3K signaling, might have implications for clinical intervention. Importantly, our data suggest that future studies should aim to exploit any potential synergies between compounds targeting these pathways that could prevent the maleficent effects associated with the toxicities of any individual compounds.

## Methods

### Cell culture

Immortalized human retinal pigmented epithelial (hTERT RPE-1) cells (gift from Dr. Gregory Pazour, University of Massachusetts Medical School, Worcester, MA) were maintained in DMEM/F-12 (Life Technologies, Carlsbad, CA, USA). Human *VHL*-deficient RCC 786-0 cells were maintained in RPMI-1640 media (Life Technologies, Carlsbad, CA, USA), 769-P (ATCC, CRL-1933) and A-498 were maintained in MEM (Life Technologies, Carlsbad, CA, USA). All cell lines were supplemented with 10% fetal bovine serum (Sigma-Aldrich, St.Louis, MO, USA). The cell lines used in this study were routinely monitored for mycoplasma and confirmed negative. In addition, human cell lines were short tandem repeat fingerprinted and validated using the Characterized Cell Line Core Facility (U.T. M. D. Anderson Cancer Center).

### Transfections and drug treatments

The image-based screen that identified NVP-BEZ235 has been previously described in^28^. All siRNA’s (siControl, siVHL, siAKT) were obtained as lyophilized powder from Dharmacon (GE Healthcare, Lafayette, CO, USA) and resuspended in 250µL of 1X siRNA buffer (Dharmacon). Cells at 70% confluence were transfected with siC, siVHL, and siAKT at 30 nm each and serum starved for 48 h to induce the formation of primary cilia. NVP-BEZ235 (Dactolisib), MLN8237 (Alisertib), MK2206 and LY294002 were obtained from Selleck Chem (Houston, TX, USA). MK2206 and LY294002 were used at final concentrations of 2 µM and 10 µM, respectively. Rapamycin (Sigma-Aldrich St.Louis, MO, USA) was used at a final concentration of 200 nM. In vitro, NVP-BEZ235 was used at 0.1 µM, 0.3 µM and 1.0 µM (solubilized in DMSO) for 48 h in serum free media. DMSO was used as a vehicle control.

### Programmatic image analysis for cilia detection

Immunofluorescence images were analyzed using a novel algorithm implemented in MATLAB (R2017a, The Mathworks). Images were loaded into MATLAB environment and separated into red, green and blue channels. The *cilia image channel* was filtered by a background image intensity threshold and by an additional cilia-object size threshold to extract the cilia objects from the background. To estimate accurately the entire cilia the thinning morphological operation was applied using the Matlab image processing library, with cilia being progressively refined to its skeleton. The *basal body channel* was filtered using a background image intensity threshold and basal-body object size threshold to programmatically extract the basal bodies from the input images. To identify cilia programmatically, minimum Euclidean distance from each cilia-object boundary points to the center of all the basal-body objects were calculated; if the minimum distance was less than 12 pixels cilia-object was identified as a cilium. If more than one basal-body-objects were determined in the vicinity, our algorithm considered this occurrence as an artifact and hence discarded these from final analysis. The number of basal bodies were obtained by counting the basal body objects detected in the input image as described above. The percentage ciliated was inferred as follows:

$${\text{Percentage ciliated}} = 100*{{\text{number of cilia}} \mathord{\left/ {\vphantom {{\text{number of cilia}} {\text{number of basal bodies}}}} \right. \kern-\nulldelimiterspace} {\text{number of basal bodies}}}.$$

### Immunoblotting

Whole cell lysates were extracted from cells (90–100% confluent) using cold 1× cell lysis buffer (20 mM Tris (pH7.5), 150 mM NaCl, 1 mM EDTA, 1 mM EGTA, 1% Triton-X-100, 2.5 mM sodium pyrophosphate and 1 mM β-glycerophosphate). 1× complete protease inhibitor cocktail (Roche, Mannheim, Germany), 1 mM sodium orthovanadate (Na_3_VO_4_), 1 mM PMSF (Sigma-Aldrich St. Louis, MO, USA) were added fresh at the time of use. The lysates were put on a rocker at 4° for 1 h, centrifuged for 10 min at full speed and the supernatant run on 4–15% SDS-PAGE gels (Bio-Rad, Hercules, CA, USA) followed by transfer to PVDF membrane. The primary antibodies used for immunoblotting were: anti-AURKA (1:1000), anti-VHL (1:1000), anti-AKT (1:2000), anti-S6 (1:3000), anti-4E-BP1 (1:2000), anti-p-AKT (S473 and T308) (1:1000), anti-p-S6 (S235/236) (1:1000), anti-p-4E-BP1 (1:1000) from Cell Signaling Technologies (Danvers, MA, USA); anti-α-Tubulin DM1A (1:5000), GAPDH (1:5000) from Santa Cruz Biotechnology (Dallas, TX, USA). Horseradish peroxidase conjugated goat anti-mouse and goat anti-rabbit antibodies (Santa Cruz Biotechnology) were used as secondary antibodies and immunoblots visualized using ECL and ECL Prime (Thermo Fisher Scientific, Waltham, MA, USA). Densitometric analysis were performed using ImageQuant (GE Healthcare Life Sciences, Pittsburg, PA, USA). Raw images of blots at different exposures are included as part of the “Supplemental Information” with the red boxes highlighting the exact blots shown in the main Figures.

### RT-PCR analysis

mRNA was isolated using Trizol from transfected and drug treated cells and further purified using phenol–chloroform extraction. Complementary DNA (cDNA) was prepared by reverse transcription (Superscript III, Life Technologies, Carlsbad, CA, USA). Gene expression was analyzed in 96-well plates by real time quantitative PCR using specific TaqMan probes (Thermo Fisher Scientific, Waltham, MA, USA) and TaqMan Fast Universal master mix on a Applied Biosystems QuantStudio 6 Flex Real-Time PCR system (Thermo Fisher Scientific, Waltham, MA, USA). The mRNA expression was determined for AURKA, VHL, and Ppia (control). For each real time reaction, the conditions were: 95 °C for 20 min followed by 40 cycles of 1 s at 95 °C and 20 s at 60 °C. All RT-PCR experiments were all done in at least triplicate and quantified using − ΔΔ cycle threshold (C_T_) method.

### Immunofluorescence

Immunofluorescence staining was performed to visualize primary cilia in hTERT-RPE1 cells. Cells were fixed with 4% paraformaldehyde for 15 min, permeabilized with 0.5% Triton-X for 10 min and then blocked for 1 h using 3.75% bovine serum albumin (BSA) (Sigma-Aldrich St. Louis, MO, USA). Primary antibodies for acetylated α-tubulin (1:4000, Clone 6-11B-1, Sigma-Aldrich, St. Louis, MO, USA) and pericentrin (1:4000, Abcam, Cambridge, MA, USA) were diluted in 3.75% BSA and cells incubated for 1 h in primary antibodies. Cells were washed 3 times (10 min/wash) before application of secondary antibodies; Alexa Fluor anti-mouse 488 and anti-rabbit 546 (Life Technologies, Carlsbad, CA, USA) for 1 h at room temperature in 3.75% BSA. Cells were washed 3 times for 10 min each and post fixed with 4% PFA, followed by counterstaining with DAPI (1:4000, Thermo Fisher Scientific, Waltham, MA, USA). Cells were visualized and imaged using a Nikon Ti2 inverted microscope with a deconvolution package (Nikon, Melville, NY, USA) at 60× magnification (secondary validation assays).

### Xenografts

A RCC mouse xenograft model using 786-0 cells was established in athymic nude mice (*Foxn1*^*nu*^, JAX Labs, Bar Harbor, ME, USA). Five million cells were injected subcutaneously into the flanks of nude mice. For the first study outlined in Fig. 5A, a week after injection, the mice were divided into two group, wherein nine mice were administered vehicle (p.o.) in the form of corn oil and 13 animals were given MLN8237 (alisertib) at 50 mg/kg (p.o.). For the second study outlined in Fig. 5D, 3 weeks after injecting cells, when tumors reached approximately 200 mm^3^, the mice were divided into four groups of which five were administered Vehicle (p.o.) in the form of corn oil, ten animals were given MLN8237 (alisertib) at 50 mg/kg (p.o.), ten animals were given NVP-BEZ235 at 45 mg/kg (p.o.) and ten more animals were given a combination of MLN8237 + NVP-BEZ235. Tumors were measured weekly using digital calipers (Thermo Fisher Scientific, Waltham, MA, USA) for 5 weeks. We were unable to measure tumors in week 4 due to unforeseen circumstances and therefore tumors were measured in week 5. The mice were sacrificed at the end of week 6 or 5 (as described in Fig. 5A,D), tumors were extracted and measured to get final tumor weights and dimensions. For tumor volume the major and minor axis of the tumor were measured, and volume calculated using the formula: length × width × width/2. Tumor tissue was fixed in formalin and stained for H&E and Ki67 and imaged on a Nikon Ci-E microscope (Nikon, Melville, NY, USA). All animal studies were performed at our AALAC approved rodent facility at Baylor College of Medicine and were conducted in compliance with Baylor College of Medicine Institutional IACUC approval. In addition, studies involving rodent models complied with the ARRIVE guidelines.

### Statistical analysis

Statistical analysis (densitometry and RT-PCR analysis) were performed using Student’s t-test (two-tailed, assuming equal variance) from at least three biological replicates. For the in vivo studies, comparisons were performed using the Kolmogorov–Smirnov test after statistical removal of outliers. The standard error of the mean (SEM) was calculated and p-values less than 0.05 were considered to be significant. All statistics were performed using Prism 8 (GraphPad, La Jolla, CA, USA) and Excel (Microsoft, Redmond, WA, USA) software.

## Supplementary Information

Supplementary Figures.
