# Author Correction: Therapeutically actionable signaling node to rescue AURKA driven loss of primary cilia in *VHL*-deficient cells

**DOI:** 10.1038/s41598-021-97270-y

**Published:** 2021-08-30

**Authors:** Pratim Chowdhury, Dimuthu Perera, Reid T. Powell, Tia Talley, Durga Nand Tripathi, Yong Sung Park, Michael A. Mancini, Peter Davies, Clifford Stephan, Cristian Coarfa, Ruhee Dere

**Affiliations:** 1 Center for Precision Environmental Health, Baylor College of Medicine, One Baylor Plaza, BCM130, Houston, TX 77030 USA; 2 Department of Molecular and Cellular Biology, Baylor College of Medicine, Houston, TX USA; 3 Center for Translational Cancer Research, Institute of Biosciences and Technology, Texas A&M College of Medicine, Houston, TX USA

Correction to:* Scientific Reports* 10.1038/s41598-021-89933-7, published online 17 May 2021

The original version of this Article contained an error in the spelling of the author Cristian Coarfa which was incorrectly given as Cristian Corfa.

The original Article has been corrected.

