# Supplementary material for: Therapeutically actionable signaling node to rescue AURKA driven loss of primary cilia in VHL-deficient cells
Source: Sci Rep. 2021 May 17;11:10461. doi: 10.1038/s41598-021-89933-7 (PMC8128866; doi:10.1038/s41598-021-89933-7)

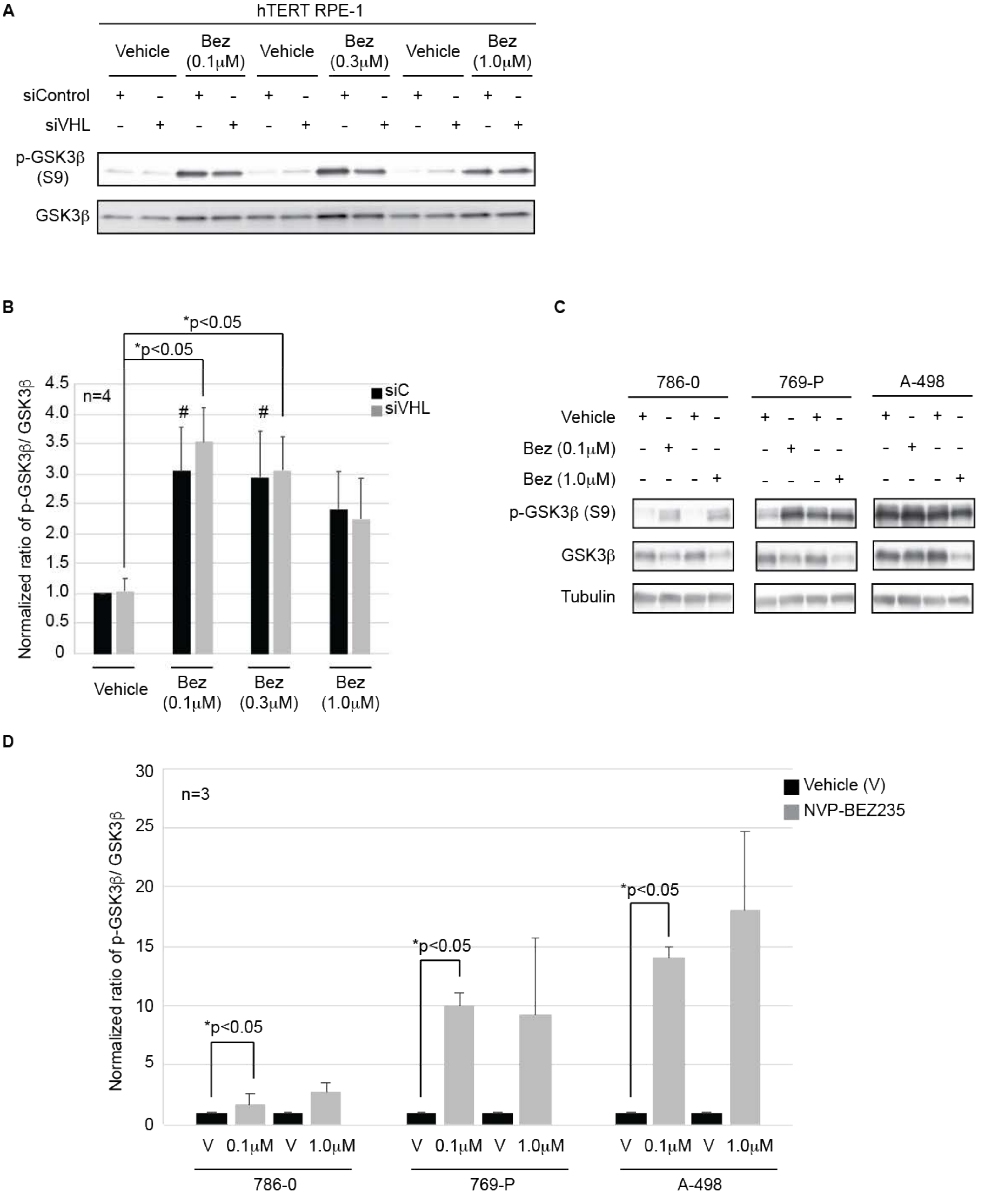

Supplemental Figure 1

A

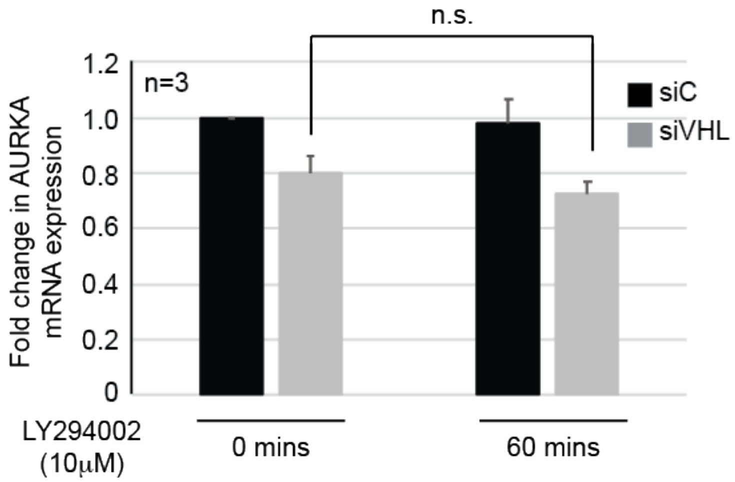
n.s.
1.2
1.0
0.8
0.6
0.4
0.2
0
n=3
siC
siVHL
Fold change in AURKA mRNA expression
LY294002 (10μM)
0 mins
60 mins

B

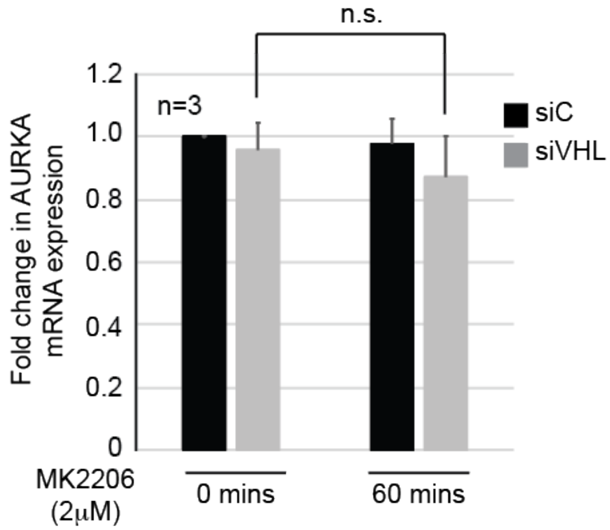
n.s.
1.2
1.0
0.8
0.6
0.4
0.2
0
n=3
siC
siVHL
Fold change in AURKA mRNA expression
MK2206 (2μM)
0 mins
60 mins

Supplemental Figure Legends

Supplemental Figure 1. **A.** *VHL*-proficient and *VHL*-deficient cells were treated with Vehicle or NVP-BEZ235 (Bez) as indicated and blotted for the antibodies shown. **B.** Densitometric analysis graphed to show the ratio of phospho-GSK3β to total GSK3β. Black bars – siC (scrambled control) and gray bars – siVHL. # denotes statistical significance compared to vehicle (DMSO) treated control (siC) ($p<0.05$), * denotes statistical significance compared to vehicle treated siVHL (p-values as indicated). **C.** Three *VHL*-null RCC cells lines (786-0, 769-P, A-498) treated with Vehicle and NVP-BEZ235 (Bez) at the indicated doses and blotted as shown. **D.** Densitometric analysis graphed to show the ratio of phospho-GSK3β to total GSK3β. Black bars – vehicle treatment and gray bars – NVP-BEZ235 treatment at the doses indicated. * denotes statistical significance compared to vehicle treated controls (p-values as indicated).

Supplemental Figure 2. **A-B.** Graphical representation of fold-change in AURKA mRNA transcript levels in hTERT RPE-1 cells transfected with siC (black bars) and siVHL (gray bars) treated with LY294002 (**A**) and MK2206 (**B**). n.s. not significant.

Figure 2A

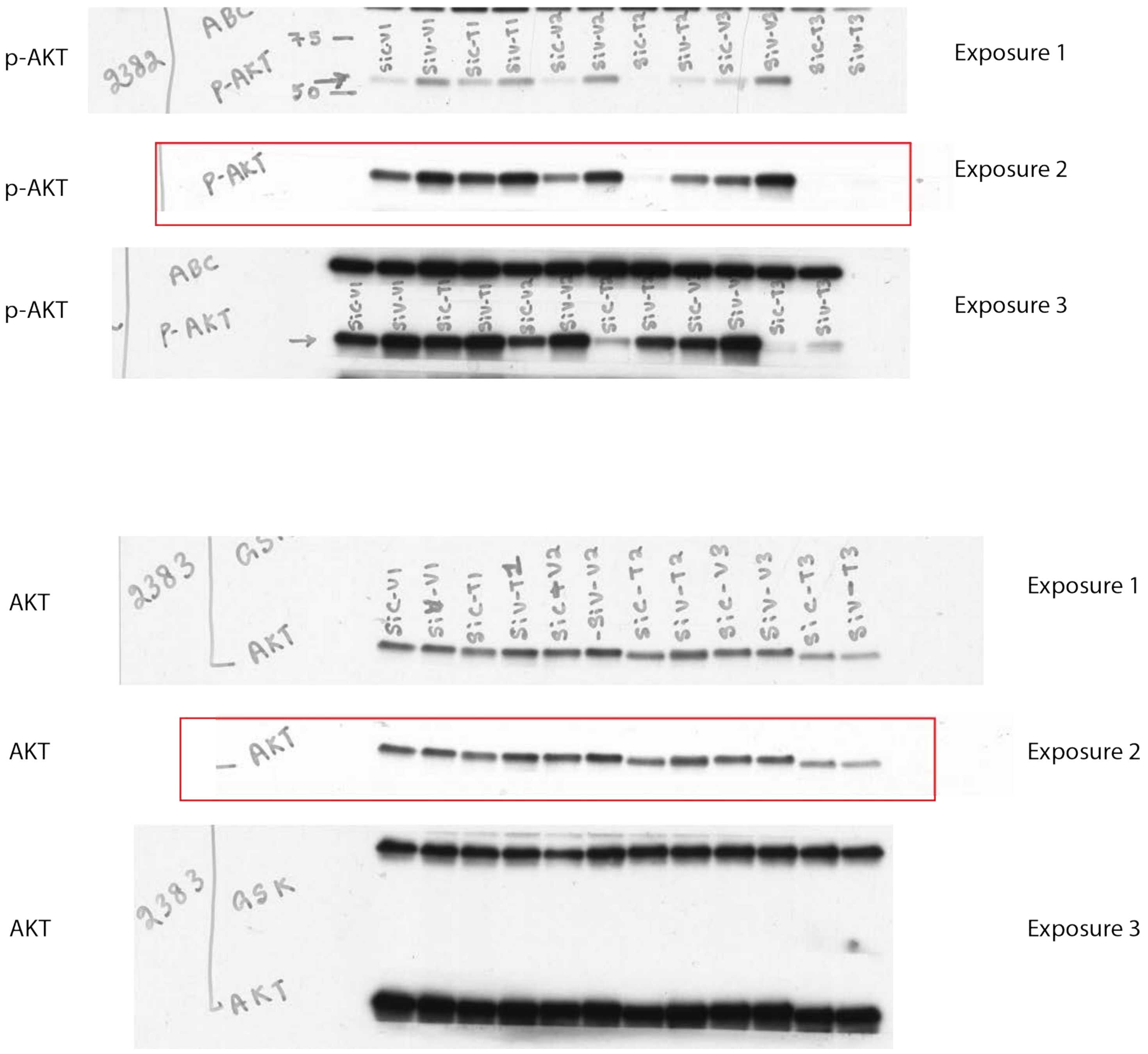
p-AKT
Exposure 1
p-AKT
Exposure 2
p-AKT
Exposure 3
AKT
Exposure 1
AKT
Exposure 2
AKT
Exposure 3

Figure 2A

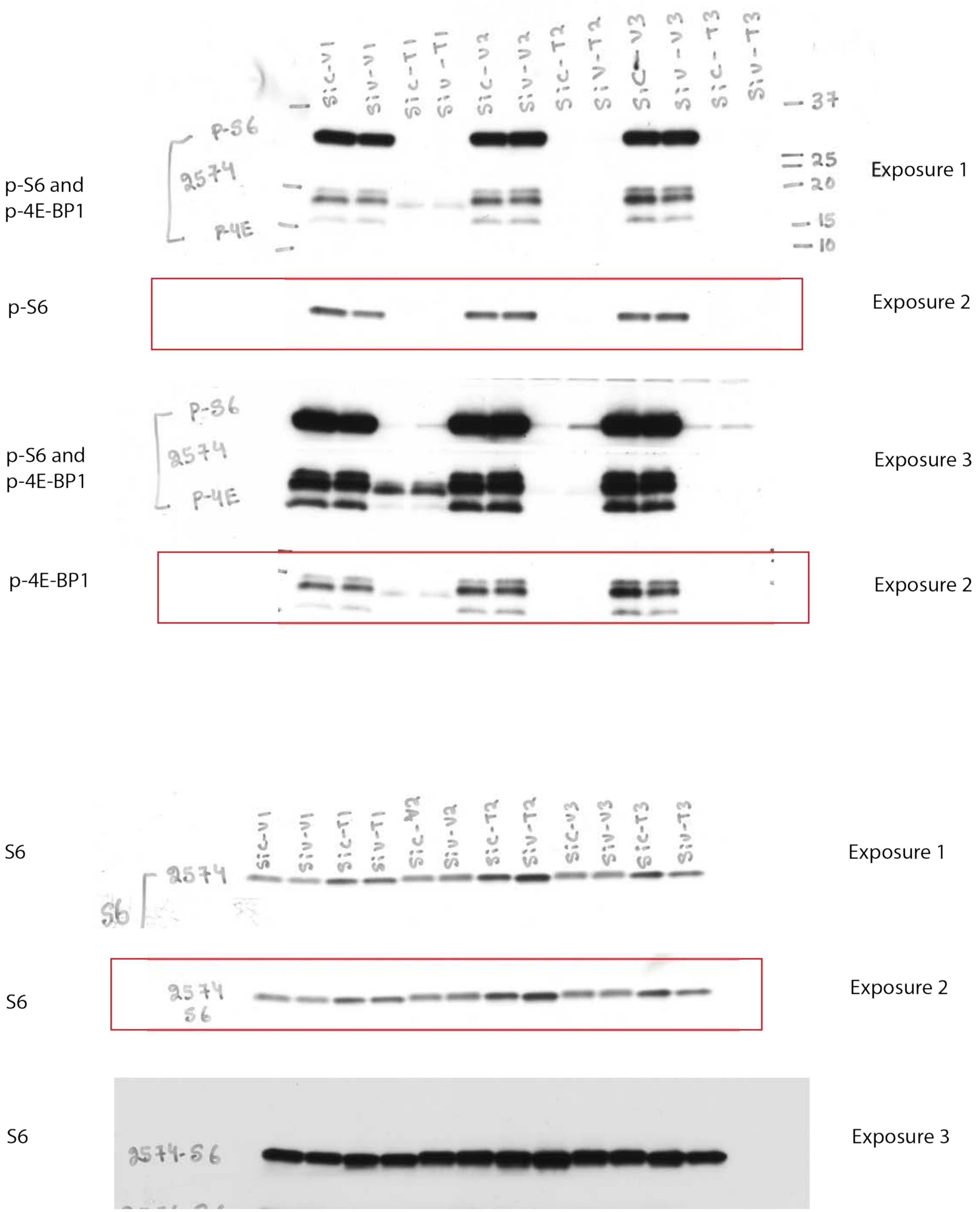

p-S6 and p-4E-BP1
Exposure 1
p-S6
Exposure 2
p-S6 and p-4E-BP1
Exposure 3
p-4E-BP1
Exposure 2
S6
Exposure 1
S6
Exposure 2
S6
Exposure 3

Figure 2A

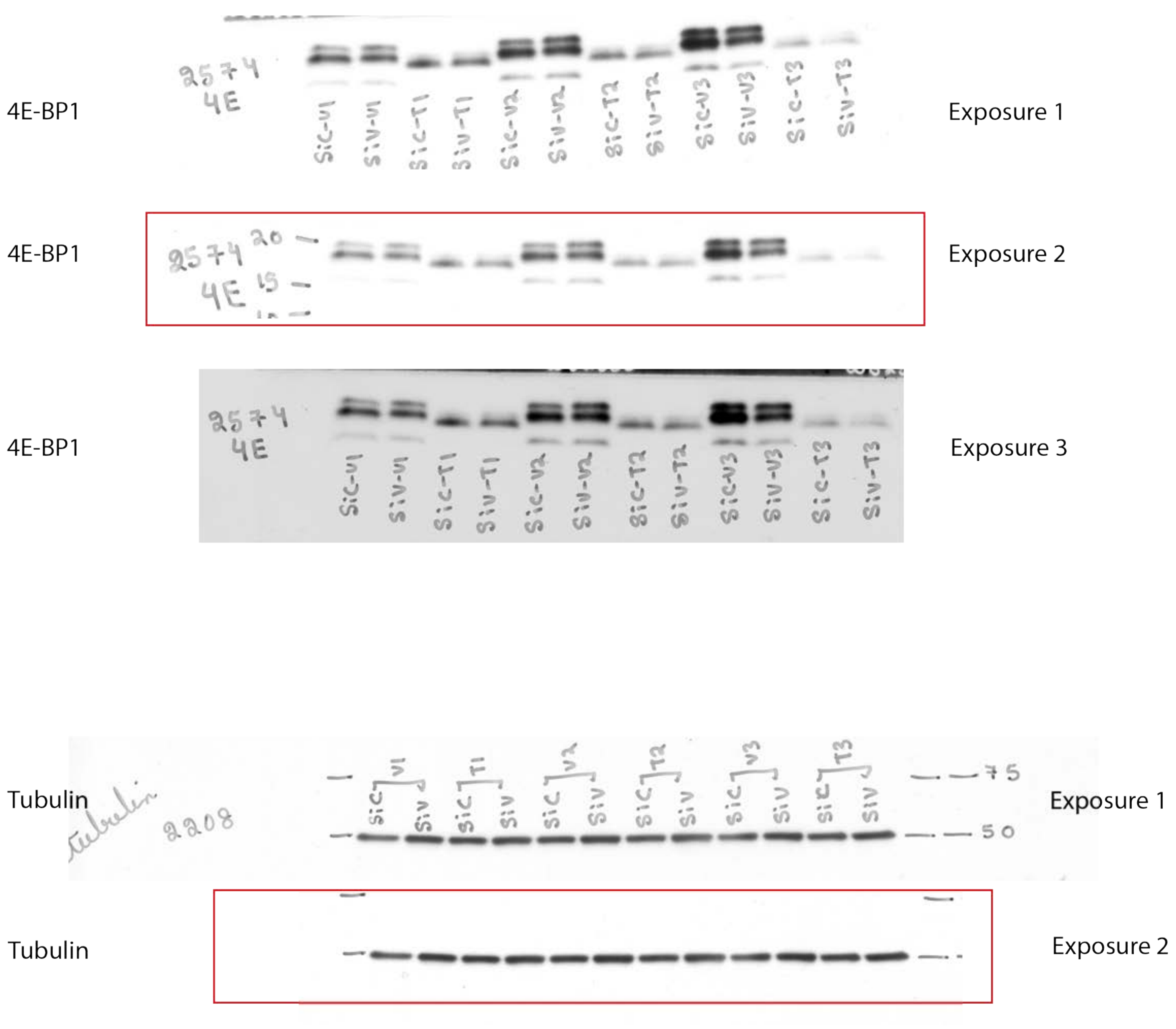
4E-BP1
Exposure 1
4E-BP1
Exposure 2
4E-BP1
Exposure 3
Tubulin
Exposure 1
Tubulin
Exposure 2

Figure 2C

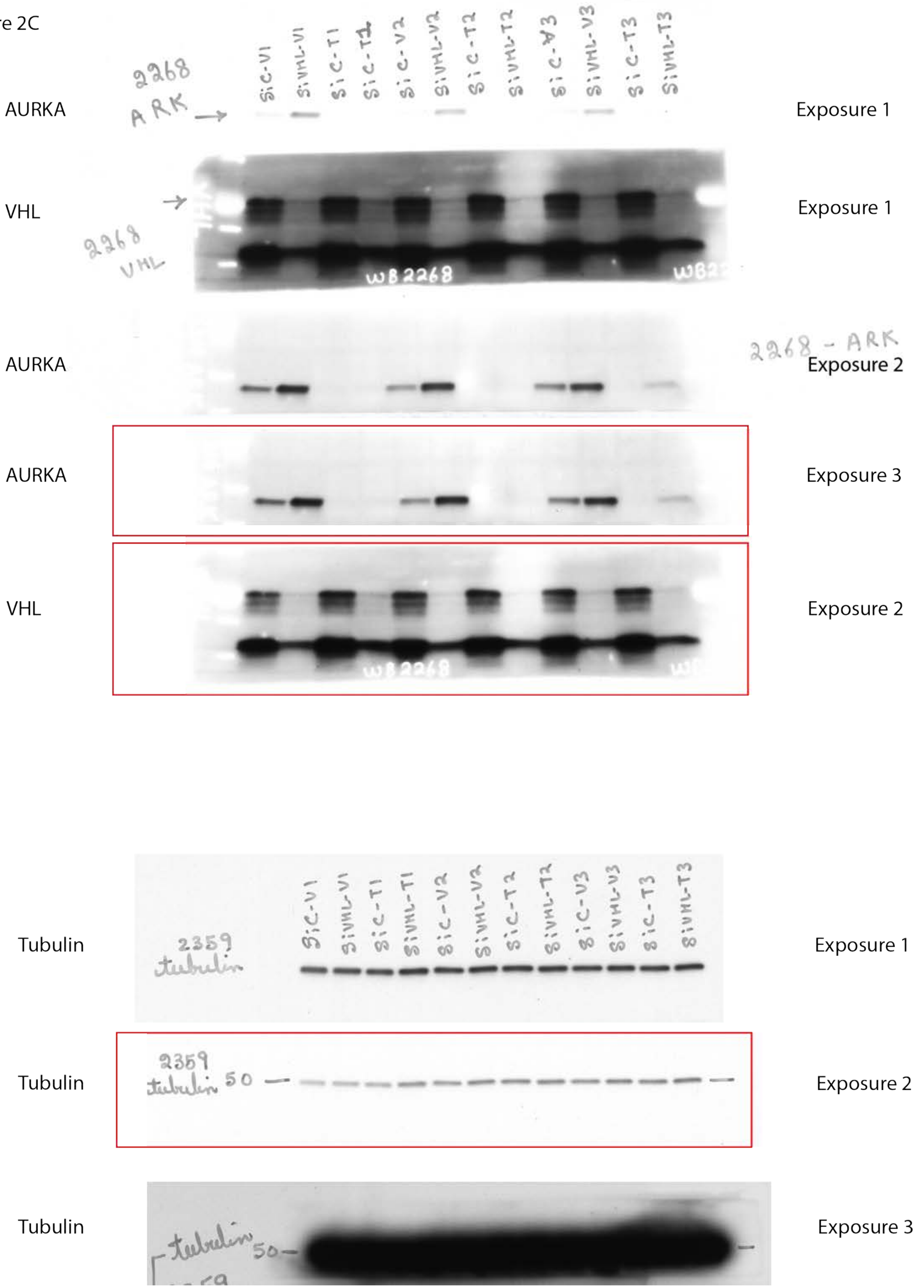

AURKA
Exposure 1
VHL
Exposure 1
AURKA
Exposure 2
AURKA
Exposure 3
VHL
Exposure 2
Tubulin
Exposure 1
Tubulin
Exposure 2
Tubulin
Exposure 3

Figure 2F

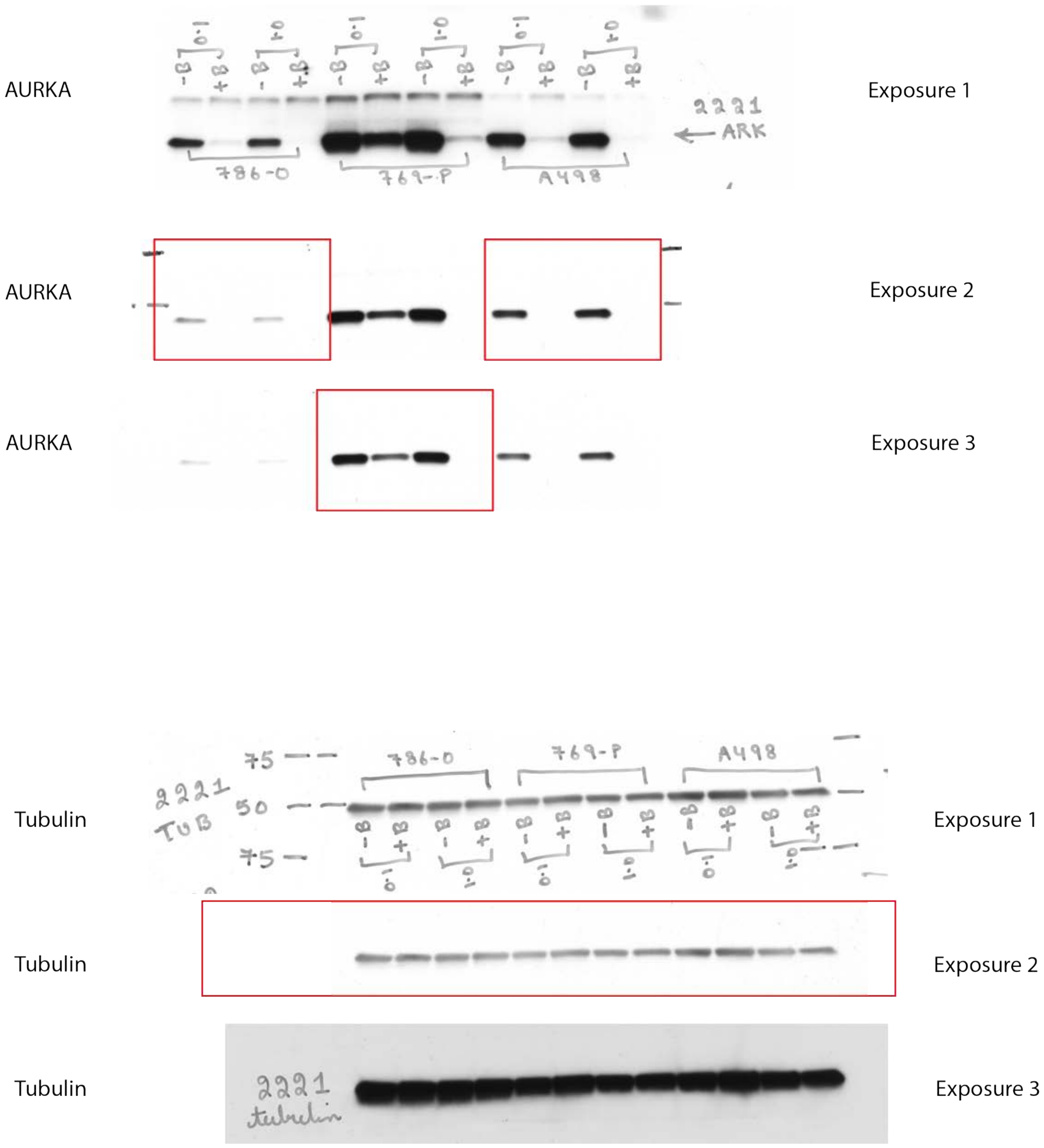

AURKA
Exposure 1
2221
ARK
786-O
769-P
A498
AURKA
Exposure 2
AURKA
Exposure 3
Tubulin
Exposure 1
2221
TUB
75
50
Tubulin
Exposure 2
Tubulin
Exposure 3
2221
tubulin

Figure 3A

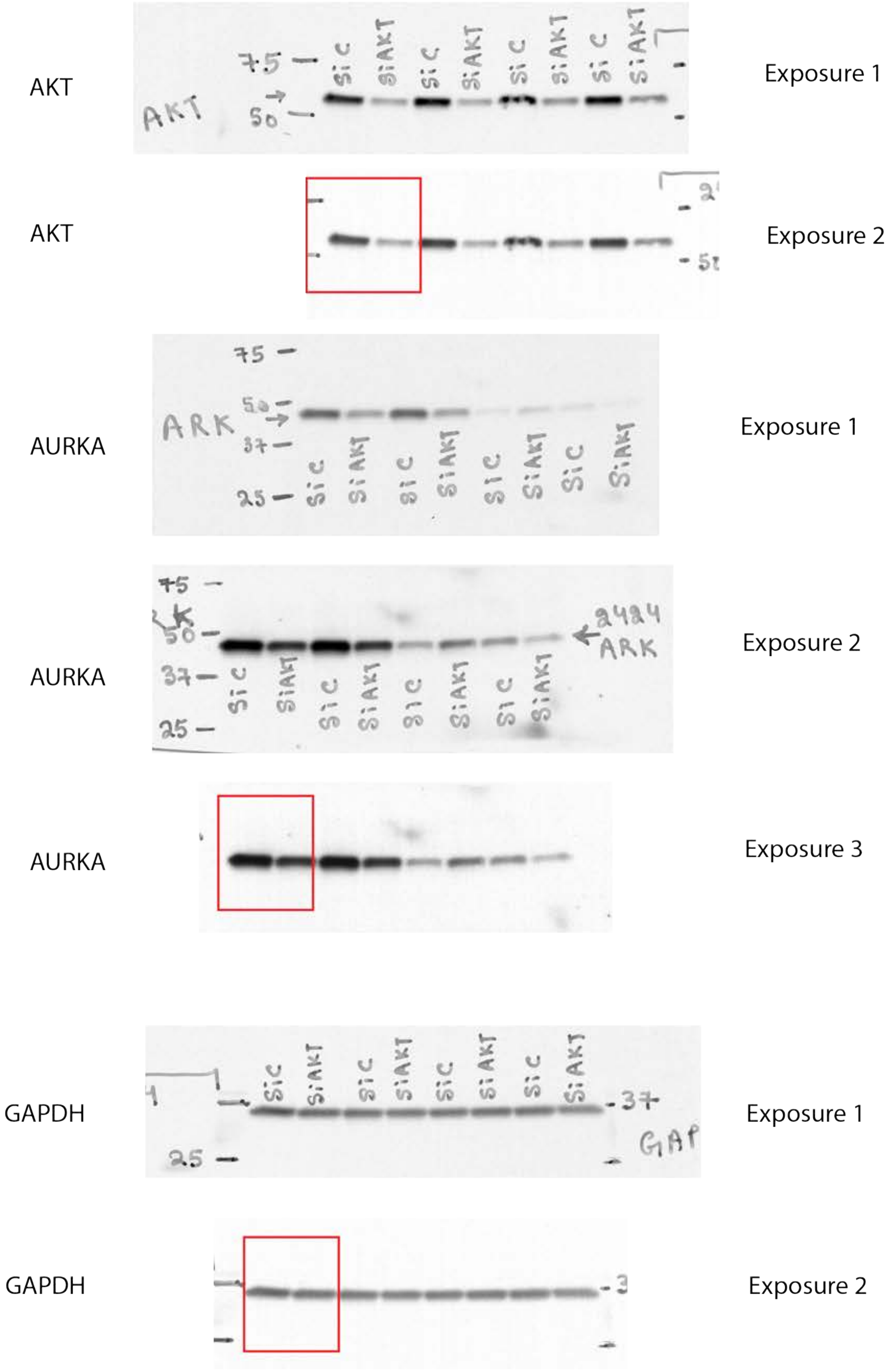
AKT
Exposure 1
AKT
Exposure 2
AURKA
Exposure 1
AURKA
Exposure 2
AURKA
Exposure 3
GAPDH
Exposure 1
GAPDH
Exposure 2

Figure 3D

AKT, GAPDH and AURKA

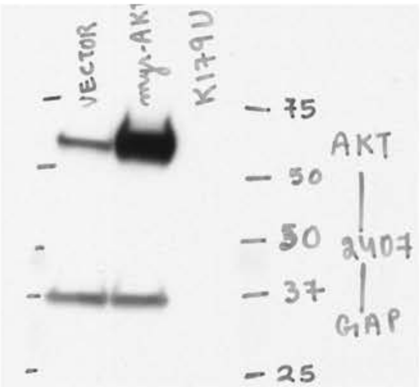

Exposure 1

AKT, GAPDH and AURKA

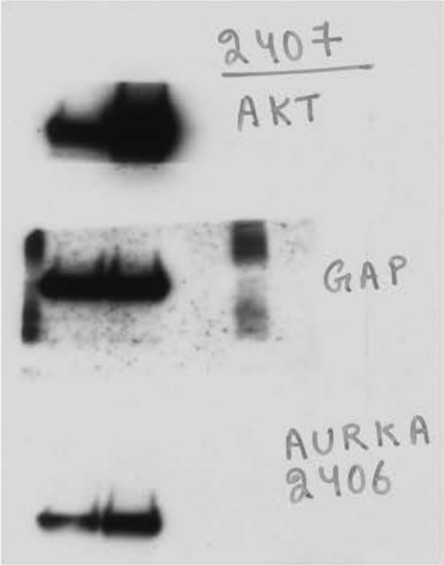

Exposure 2

AKT

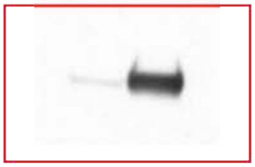

Exposure 3

GAPDH

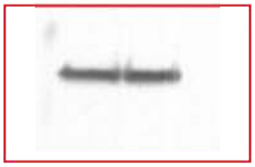

Exposure 3

AURKA

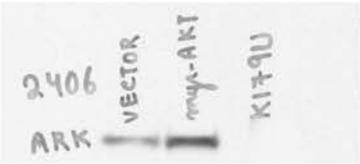

Exposure 2

AURKA

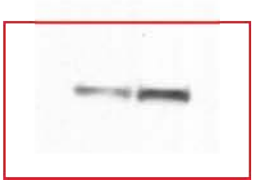

Exposure 3

Figure 3E

AKT
GAPDH
p-AKT
AURKA

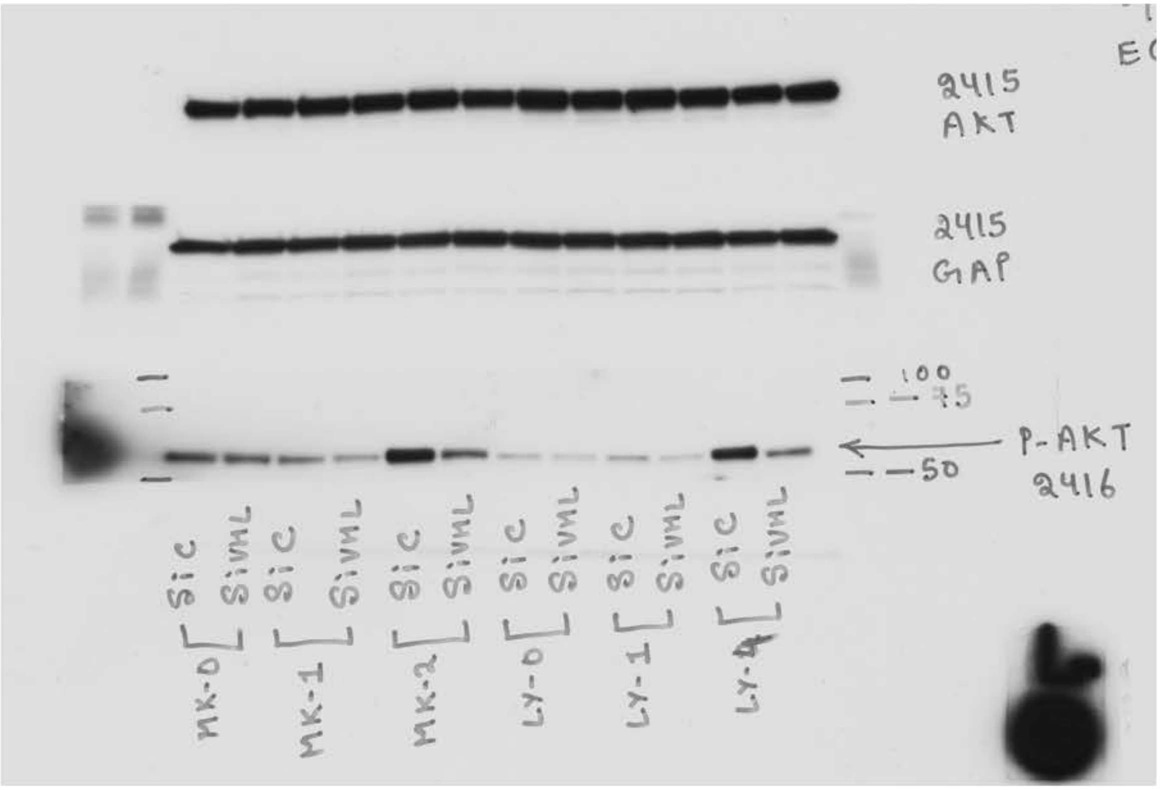

Exposure 1

AKT
GAPDH
p-AKT
AURKA

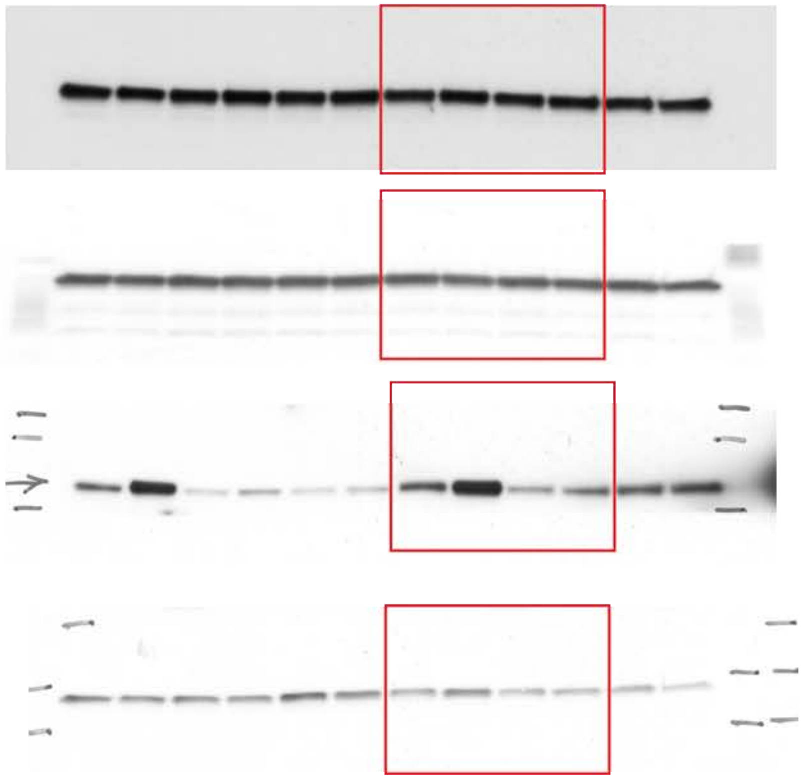

Exposure 2

Figure 3G

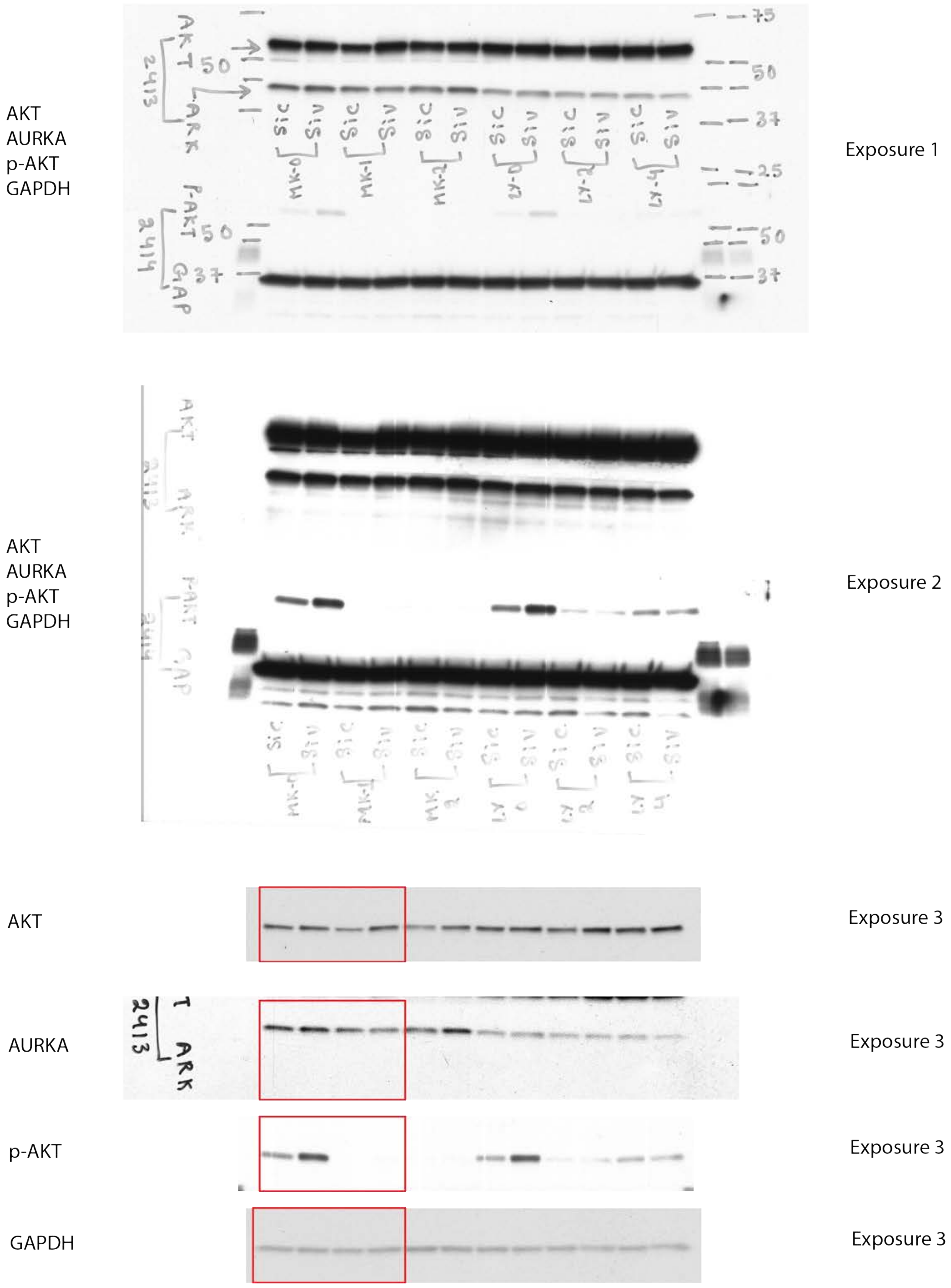
AKT
AURKA
p-AKT
GAPDH
Exposure 1
AKT
AURKA
p-AKT
GAPDH
Exposure 2
AKT
Exposure 3
AURKA
Exposure 3
p-AKT
Exposure 3
GAPDH
Exposure 3

Figure 3I

AURKA
S6
p-S6
Tubulin
VHL

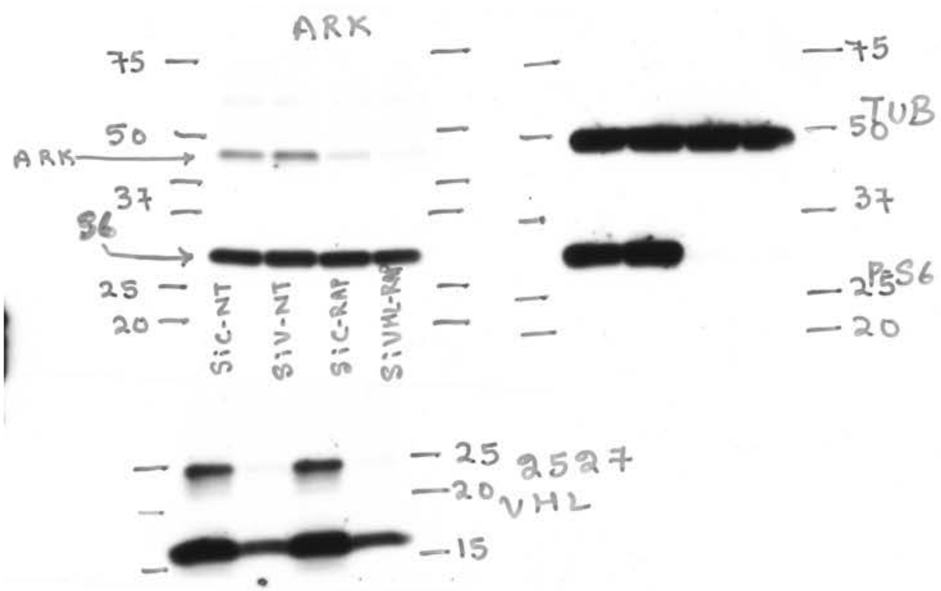

Exposure 1

AURKA
S6

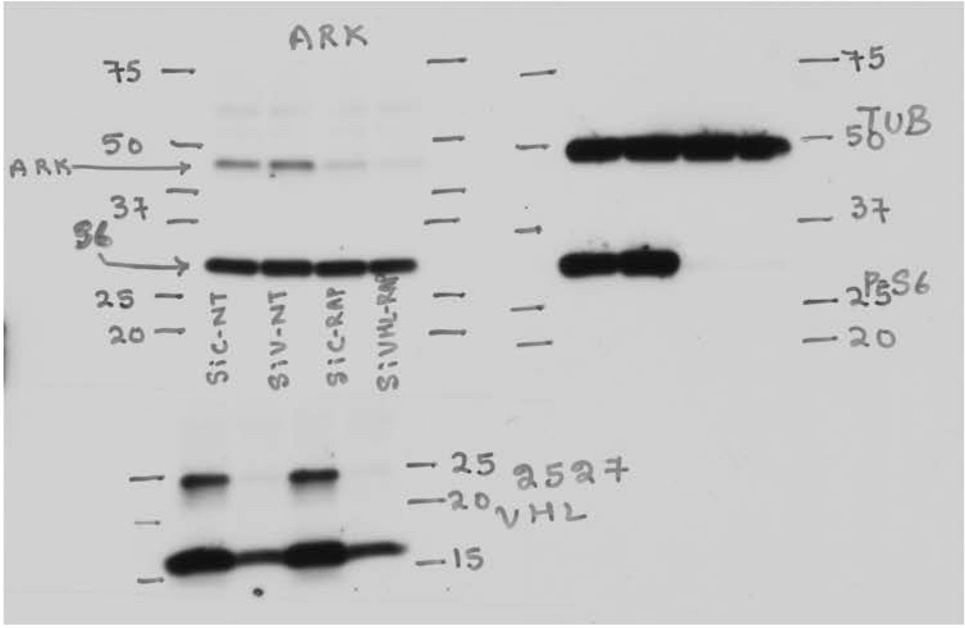

Exposure 2

AURKA

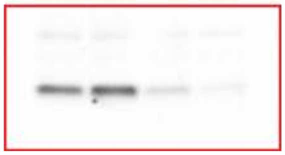

Tubulin

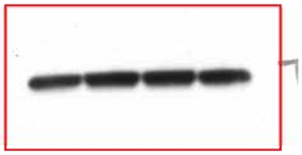

pS6

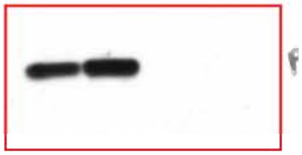

Exposure 3

S6

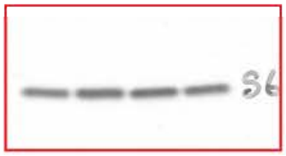

VHL

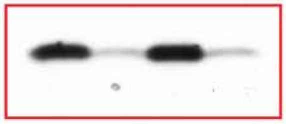

Figure 4C

AURKA
VHL
AKT
GAPDH

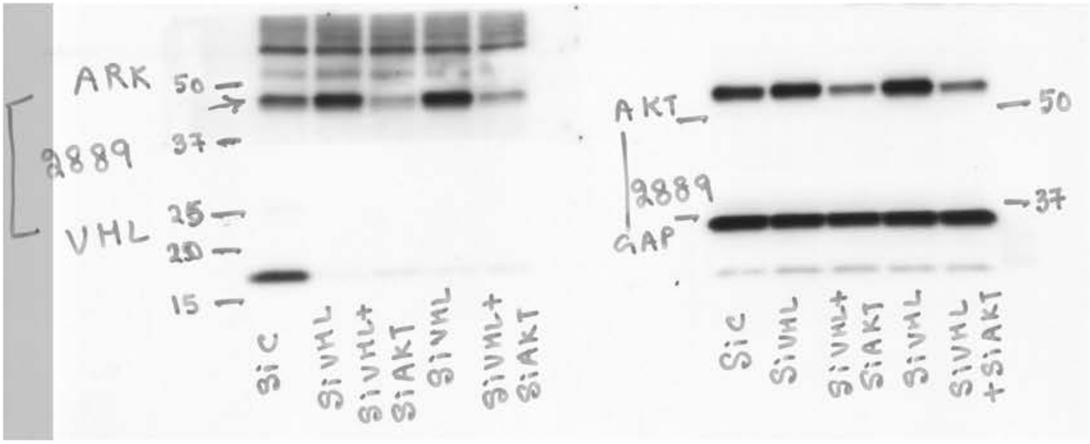

Exposure 1

AURKA
VHL

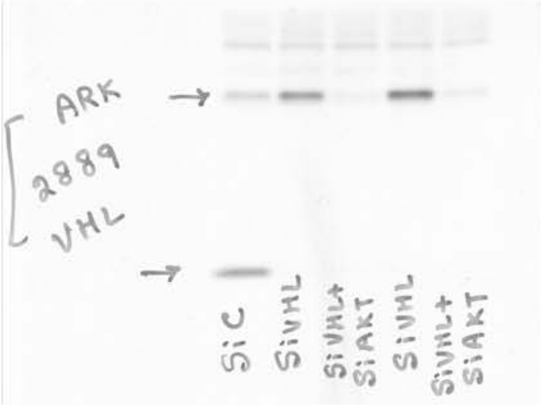

Exposure 2

AURKA
VHL

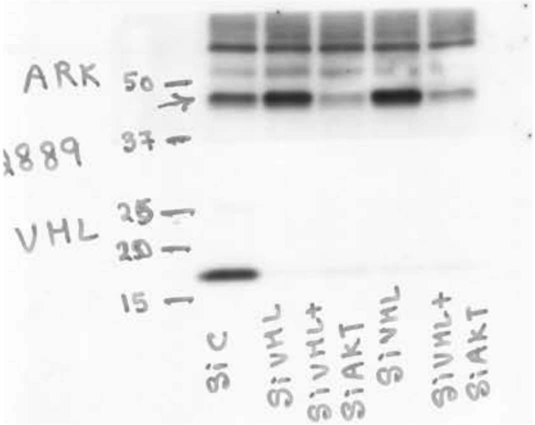

Exposure 3

AURKA

VHL

AKT

GAPDH

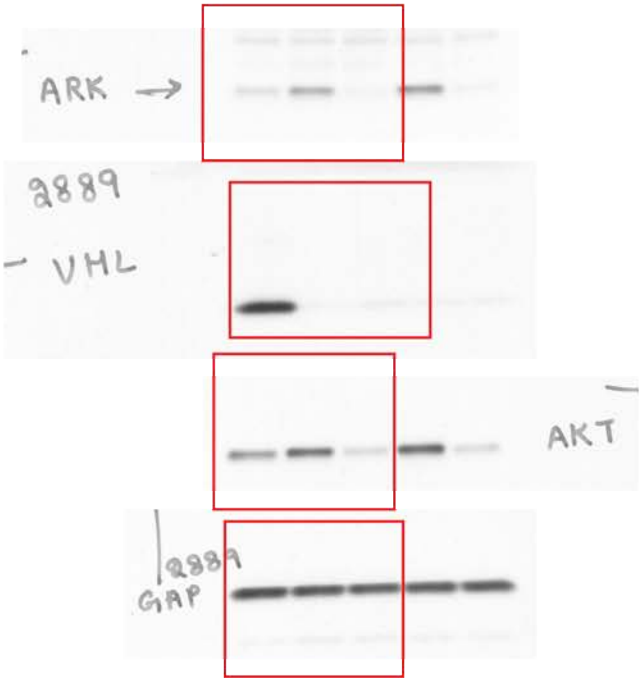

Exposure 4

Supplementary Figure 1A

p-GSK3β — Exposure 1

p-GSK3β — Exposure 2

GSK3β — Exposure 1

GSK3β — Exposure 2

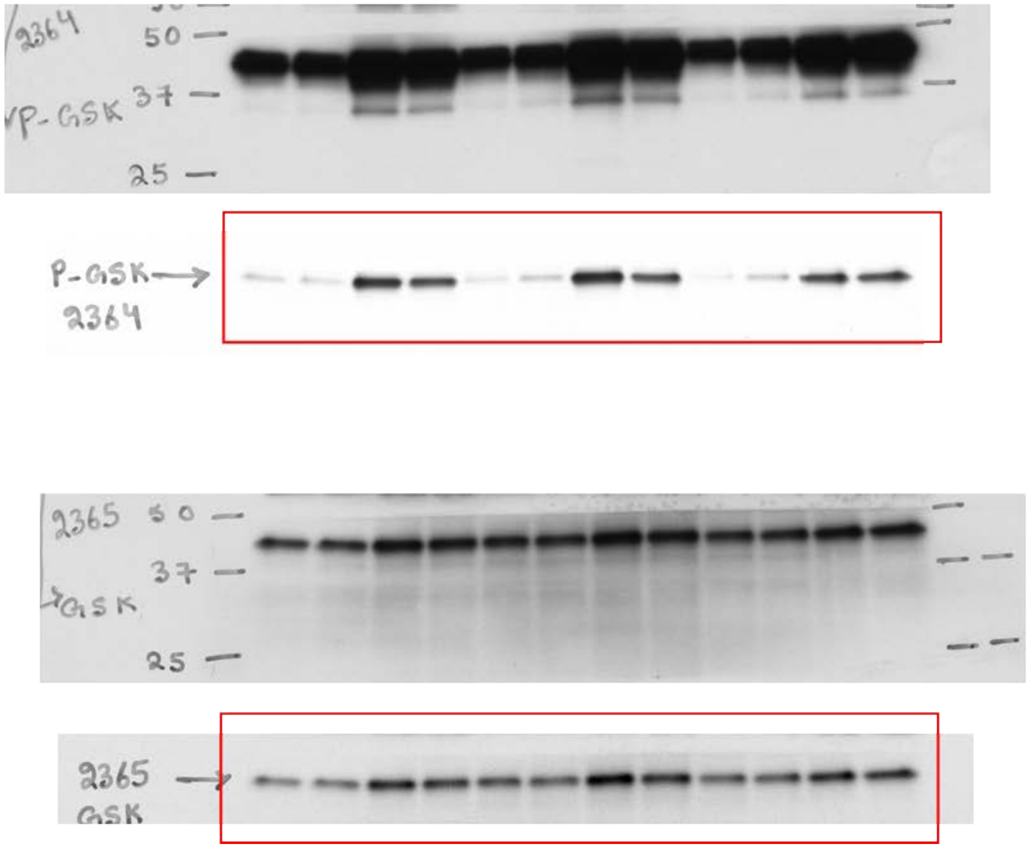

Supplementary Figure 1C

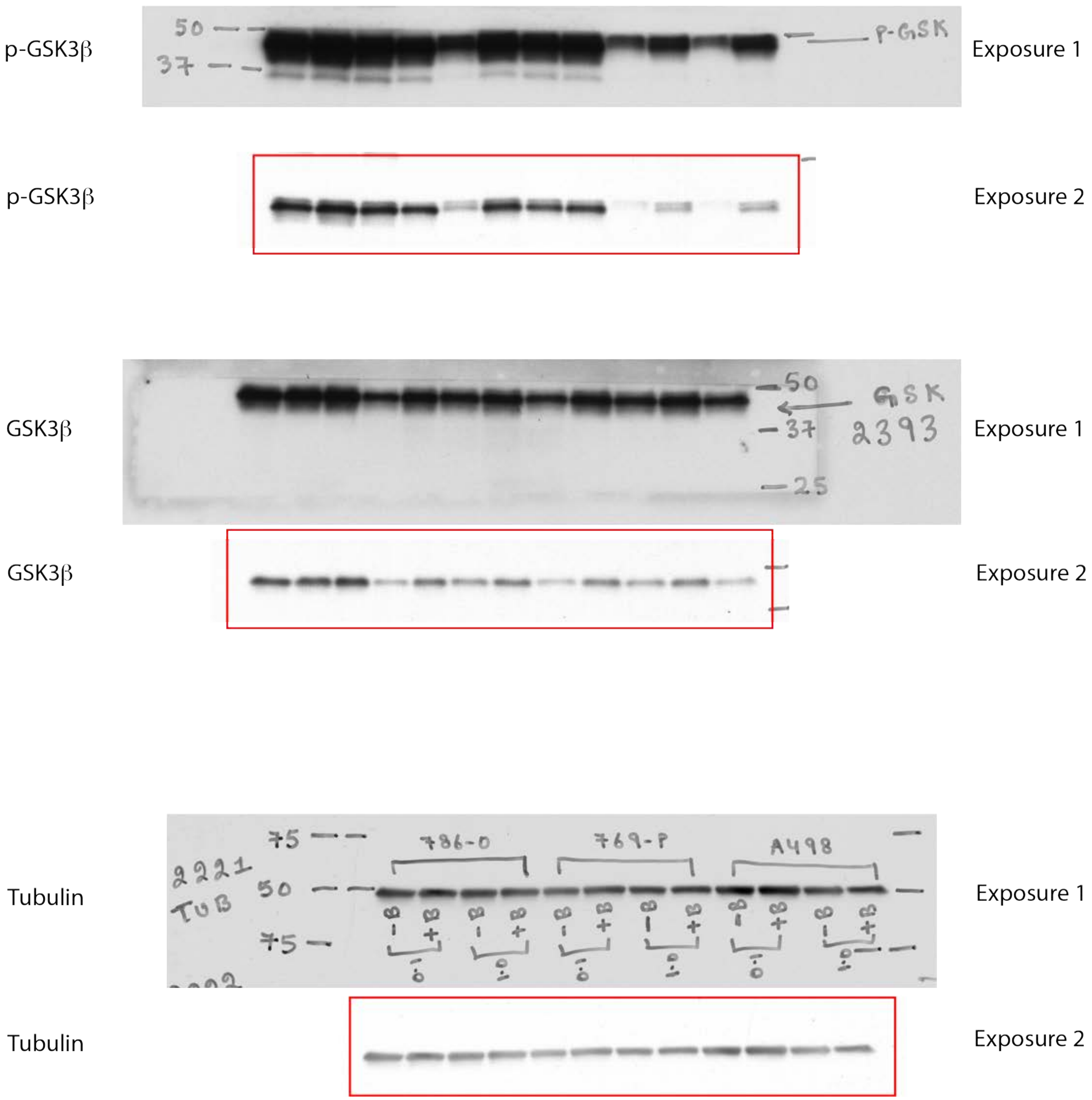

Supplement: Supplementary file 1 — Supplementary Figures. [file 41598_2021_89933_MOESM1_ESM.pdf]
